# Technologies for healthy lifestyle in people with HIV: a systematic review

**DOI:** 10.1590/1980-220X-REEUSP-2024-0396en

**Published:** 2025-05-09

**Authors:** Gilmara Holanda da Cunha, Yrene Esperanza Urbina Rojas, Maiara Bezerra Dantas, Maria Elisa Curado Gomes, Larissa Rodrigues Siqueira, Marina Soares Monteiro Fontenele

**Affiliations:** 1 Universidade Federal do Ceará, Fortaleza, CE, Brazil.; 2 Universidad Nacional de Tumbes, Tumbes, Peru.

**Keywords:** HIV, Acquired Immunodeficiency Syndrome, Healthy Lifestyle, Educational Technology, Nursing

## Abstract

**Objective:**

to analyze clinical trials that assessed the effectiveness of technologies for healthy lifestyles in people with HIV.

**Method:**

A systematic review, conducted in five databases, with association of controlled descriptors. Complete and electronically available randomized controlled clinical trial articles, without language or date restrictions, involving technologies for healthy lifestyles in people with HIV over 18 years of age were included. Studies involving children, adolescents, pregnant women and repeated articles were excluded. The Risk-of-Bias Tool for Randomized Trials and the Assessment of Multiple Systematic Reviews were used to assess bias and review quality.

**Results:**

A total of 2,933 articles were identified and eight were selected. The technologies were mobile applications, booklets, motivational interviewing and telephone interventions, focusing on self-care, antiretroviral adherence, stress management, fatigue and depression, and encouraging reduction in smoking, alcohol and drug use.

**Conclusion:**

The technologies were classified as soft-hard and hard, and improved the lifestyle of people with HIV. Registration in the International Prospective Register Systematic Reviews (PROSPERO): CRD42023422772.

## INTRODUCTION

Antiretroviral therapy (ART) has reduced mortality from acquired immunodeficiency syndrome (AIDS) and infection by the human immunodeficiency virus (HIV) has become a chronic condition, with increased survival and, concomitantly, a higher incidence of diseases not related to AIDS^(1)^. Disease control through antiretroviral agents is part of the 95-95-95 target, which aims to ensure that, by 2030, 95% of people living with HIV (PLWHIV) know their serological status, 95% of them are on antiretroviral treatment and 95% have their viral load suppressed^(2)^.

Care for PLWHIV that was previously focused on opportunistic infections has been transferred to other health problems that affect the general population^(3)^. Thus, it is considered that adopting practices for a healthy lifestyle, through adequate nutrition, physical exercise, non-use of legal and illegal drugs, stress control, and adherence to antiretroviral agents and other medications, is an essential conduct to improve the quality of life of people with HIV^(4)^. However, there is a lack of encouragement to promote self-care for a healthy lifestyle among these patients^(5)^.

Studies show that, although PLWHIV have an increase in survival with ART, chronic non-communicable diseases (NCDs) occur more frequently in this population, especially chronic obstructive pulmonary disease, ischemic heart disease, mental illnesses, and kidney and liver dysfunctions^(6,7)^. Lifestyle is a factor in the emergence and maintenance of NCDs, and changes in this aspect represent an important intervention to prevent comorbidities and combat diseases^(8)^. Care tools are needed for ongoing counseling of PLWHIV as well as health education interventions that encourage active patient participation in the therapeutic plan^(9,10)^.

In view of this, technologies emerge as tools that facilitate care, capable of disseminating accurate information and indispensable knowledge^(11)^. Studies show that technologies can help PLWHIV improve their lifestyle by changing habits^(9,10)^. Advances in technology provide innovations in the health area, raising awareness among PLWHIV about self-care, contributing to access to information and directing those who need healthcare services^(5)^.

Health technologies are considered manuals, booklets, folders, e-books, educational programs and software^(12)^, which play a crucial role in the development of HIV prevention actions and in patient monitoring^(12)^. Health technologies can be classified as soft (technology of relationships, production and communication, which involve welcoming, bonding and listening); soft-hard (well-structured knowledge in the health process, formulation of educational materials and application of theories); and hard (technological equipment, instruments, standards and software)^(13)^. Technologies can assist nurses and other members of the multidisciplinary health team in assistance and guidance activities, in order to contribute to self-care and adherence to a healthy lifestyle by PLWHIV^(9)^. However, before they can be used by healthcare professionals and patients, their effectiveness must be assessed.

Effective health technologies for promoting healthy lifestyles help both promote care by professionals and increase autonomy and adherence to such care by PLWHIV. Assessing whether these technologies are effective can favor evidence-based practice, since there are several challenges to implementing care in this population, such as difficulty in accessing healthcare services due to stigma resulting from the disease and fear of disclosing serological status^(1,11)^, low functional health literacy^(3)^, and low education and income^(2,3,9,11)^. All of these aspects can negatively impact healthcare and lifestyle.

Given the above, this study was guided by the following research question: how effective are technologies used to promote healthy lifestyles in PLWHIV? Its objective was to analyze clinical trials that assessed the effectiveness of technologies for healthy lifestyles in people with HIV.

## METHOD

### Study Design

This is a systematic review, with a quantitative, descriptive approach and without meta-analysis, carried out in six stages: 1. Guiding question elaboration; 2. Protocol formation; 3. Generation of a list of relevant studies; 4. Study selection for analysis; 5. Study quality and data extraction assessment; 6. Manuscript synthesis and writing^(14)^.

The Preferred Reporting Items for Systematic Reviews and Meta-Analyses (PRISMA)^(15)^ recommendations were followed, and the systematic review was registered in the International Prospective Register of Systematic Reviews (PROSPERO) in 2023, under registration CRD42023422772.

### Study Place and Period

The survey of articles in databases took place between October 2023 and January 2024 by researchers located in Fortaleza, Ceará, Brazil, and in Tumbes, Peru.

### Eligibility Criteria

The research question was formulated according to the PICO strategy, an acronym for Patient (people with HIV), Intervention (effectiveness of technologies for healthy lifestyle), Comparison (not applied in the study, as the objective was not to compare interventions) and Outcome (improvement of lifestyle). The systematic review had as its guiding question: how effective are the technologies used for healthy lifestyle in PLWHIV?

Randomized clinical trial studies, classified as level of evidence II, which are randomized and controlled clinical trials^(16)^, in addition to complete articles available electronically, without language or publication date restrictions, involving technologies for healthy lifestyle in PLWHIV over 18 years of age, were included. Studies with children, adolescents and pregnant women, in addition to repeated articles, which were counted only once, were excluded.

### Sources of Information and search Strategies in Databases

The articles were selected from five databases: Medical Literature Analysis and Retrieval System Online (MEDLINE); Scopus; Embase; Web of Science; and Latin American and Caribbean Literature in Health Sciences (LILACS). The databases were selected based on the guiding question, types of studies desired and visibility in the health area.

The descriptors “HIV”, “HIV Infections”, “Acquired Immunodeficiency Syndrome”, “Technology”, “Technologies”, “Life Style” and “Healthy Lifestyle” were used, all from the Health Sciences Descriptors (DeCS) of the Virtual Health Library and the Medical Subject Headings (MeSH) of the National Library of Medicine. The descriptors were readjusted according to the search location: entered in English, selecting the Boolean operators AND and OR in MEDLINE, Scopus and Web of Science; entered in Portuguese, English and Spanish, and the Boolean operator AND in LILACS; entered in English, selecting the option with Boolean AND in Embase. Table 1 describes the search strategies and the number of articles found.

**Table 1:** Search strategies for articles in databases – Fortaleza, CE, Brazil, 2024.

Databases	Crossings in databases	Number of articles
MEDLINE	(“Acquired Immunodeficiency Syndrome”[Mesh] OR “HIV”[Mesh]) AND “Technology”[Mesh]	1,371
Scopus	(Technologies OR Technology) AND Lifestyle AND HIV	53
Embase	(‘Human Immunodeficiency Virus’/exp OR ‘Human Immunodeficiency Virus’ OR ‘Acquired Immune Deficiency Syndrome’/exp OR ‘Acquired Immune Deficiency Syndrome’) AND (‘Technology’/exp OR Technology) AND (‘Lifestyle’/exp OR Lifestyle)	218
Web of Science	(“HIV Infections” OR “Acquired Immunodeficiency Syndrome”) AND (Technology OR Technologies)	841
LILACS	(HIV) AND (Technology) AND (“Healthy Lifestyle”) OR (“Life Style”)	196

### Process of Extracting and Analyzing Information from Selected Studies

Article selection and analysis were carried out between February and May 2024 by two independent reviewers, and a third was used to define cases of disagreement between the others. After searching the electronic databases, the articles were exported to Rayyan, an online tool for constructing systematic reviews^(17)^. Initially, duplicate studies were removed and then all titles and abstracts were read to identify relevant studies, considering the inclusion and exclusion criteria. Articles in which these criteria were unclear were read in full. Subsequently, eligibility was assessed by reading the selected studies in full. Data were extracted from articles and organized into clinical records with information on title, authorship, year of publication, country of study, objectives, sample, intervention and control groups, outcomes, and biases.

### Assessment of Study Biases

To assess research biases, the Risk-of-Bias Tool For Randomized Trials (RoB 2.0)^(18)^ was used. To assess systematic review quality, the Assessment of Multiple Systematic Reviews (AMSTAR) was used^(19)^.

The eight articles that addressed the research question were analyzed using an organized approach to weigh study rigor and characteristics, observing methodological development, intervention, sample, results, conclusion and possible biases of the research. The risk of bias of studies was assessed using the RoB 2.0 tool in five domains: 1. Bias arising from the randomization process; 2. Bias due to deviations from intended interventions; 3. Bias due to missing outcome data; 4. Bias in measurement of the outcome; and 5. Bias in selection of the reported result. Each domain was assessed as low risk of bias, high risk of bias or some concern^(18)^.

AMSTAR assessed systematic review quality. It is a 16-item instrument that corresponds to the minimum requirements for a systematic review^(19)^: 1. Did the research questions and inclusion criteria for the review include the components of PICO? 2. Did the report of the review contain an explicit statement that the review methods were established prior to the conduct of the review and did the report justify any significant deviations from the protocol? 3. Did the review authors explain their selection of the study designs for inclusion in the review? 4. Did the review authors use a comprehensive literature search strategy? 5. Did the review authors perform study selection in duplicate? 6. Did the review authors perform data extraction in duplicate? 7. Did the review authors provide a list of excluded studies and justify the exclusions? 8. Did the review authors describe the included studies in adequate detail? 9. Did the review authors use a satisfactory technique for assessing the risk of bias (RoB) in individual studies that were included in the review? 10. Did the review authors report on the sources of funding for the studies included in the review? 11. If meta-analysis was performed did the review authors use appropriate methods for statistical combination of results? 12. If meta-analysis was performed, did the review authors assess the potential impact of RoB in individual studies on the results of the meta-analysis or other evidence synthesis? 13. Did the review authors account for RoB in individual studies when interpreting/discussing the results of the review? 14. Did the review authors provide a satisfactory explanation for, and discussion of, any heterogeneity observed in the results of the review? 15. If they performed quantitative synthesis did the review authors carry out an adequate investigation of publication bias (small study bias) and discuss its likely impact on the results of the review? 16. Did the review authors report any potential sources of conflict of interest, including any funding they received for conducting the review?

All items are answered with “yes” for positive results, “no” in cases where there was no information available or the evaluator felt that they could not opt for the benefit of the doubt, or “partially yes” when it was considered valid to indicate partial adherence to the domain. Seven of the 16 items are critical (1, 4, 7, 9, 11, 13 and 15). At the end of assessment, the review is classified as critically low (more than one critical failure), low (one critical failure), moderate (more than one non-critical failure) and high (no failure or one non-critical failure)^(19)^. AMSTAR was applied independently by two evaluators, and differences in assessments were discussed and agreed upon by consensus. Finally, the findings of articles were discussed according to scientific literature.

### Ethical Aspects

As for ethical aspects, the writings of articles and copyrights were respected, without modification of the identified content, for the benefit of this study proposed by the authors.

## RESULTS

**Figure 1:**
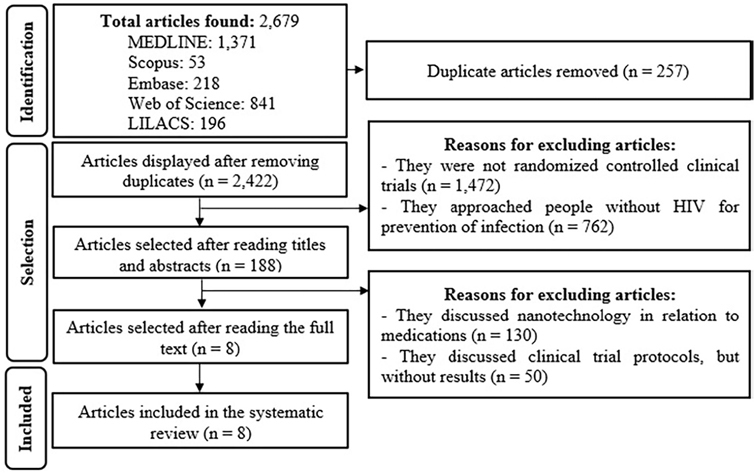
Study selection flowchart, adapted from the Preferred Reporting Items for Systematic Reviews and Meta-Analyses. Fortaleza, CE, Brazil, 2024.

A total of 2,679 articles were identified and, after analysis, a total of eight comprised the study. Figure 1 shows the flowchart with the number of articles selected.

The year of publication of articles varied between 2006 and 2023, with three published in the United States, two in Brazil, one in Canada, one in Vietnam, and one in China^(3,11,20,21,22,23,24,25)^. Article characterization regarding title, country of study, objectives, sample, groups and outcomes is found in Table 2.

**Table 2:** Studies on technologies for healthy lifestyles in people with HIV according to title, country of study, objectives, sample, groups and outcomes – Fortaleza, CE, Brazil, 2024.

Study title/country	Objectives	Sample	Intervention group	Control group	Outcomes
Using motivational interviewing to promote adherence to antiretroviral medications: a randomized controlled study^(20)^/United States	Assess motivational interviewing to improve adherence to ART in PLWHIV.	Total: 247 IG: 125 CG: 122	In-person motivational interview, applied by a nurse in five sessions, lasting 20-90 minutes, for three months in person and by telephone.	Institutional standard care.	IG was superior to CG in adherence to ART six months after the start of the intervention (p < 0.001).
A feasibility study to develop and test a cognitive behavioral stress management mobile health application for HIV-related fatigue^(21)^/United States	Determine the feasibility and acceptability of the CBSM mHealth smartphone application to address and manage stress and fatigue in PLWHIV.	Total: 30 IG: 15 CG: 15	They downloaded and used the CBSM mHealth app on their smartphones, and were asked to complete one module per week.	Downloaded the Lifesum app, which is a generic healthy lifestyle app, and used it once a week.	IG: superior reduction in fatigue than CG: (mean difference: 1.6; 95% CI: 0.3-2.8). IG: superior decrease in depression up to the 5^th^ week (mean difference: 9.7; 95% CI: 1.1-18.4).
Knowledge, attitude and practice of people with HIV regarding a healthy lifestyle: clinical trial^(3)^/Brazil	Assess the effectiveness of a booklet on knowledge, attitude and practice for healthy lifestyle in PLWHIV.	Total: 144 IG: 70 CG: 74	Received the booklet in his office, read it completely and took it home to read every 15 days for six months.	Usual service care.	The educational booklet improved knowledge, attitude and practice about healthy lifestyle among PLWHIV.
Mobile health technology for improving symptom management in low income persons living with HIV^(23)^/Canada	Examine the impact of an mHealth application (mVip) with self-care strategies for symptoms of PLWHIV.	Total: 76 IG: 40 CG: 40	They logged into mVip at least once a week to assess symptoms and receive self-care guidance.	They downloaded the mVIP, but did not receive self-care strategies.	IG: improvement in 12 of the 13 symptoms of HIV infection and in adherence to ART (p = 0.017) compared to CG.
Booklet for healthy lifestyle in people with HIV: a clinical trial^(11)^/Brazil	Assess the effectiveness of an educational booklet to promote a healthy lifestyle and adherence to ART in PLWHIV.	Total: 174 IG: 70 CG: 74	Routine medical consultation, reading the booklet at the clinic, taking the booklet home to read fortnightly for six months.	Routine medical consultation.	At baseline, the lifestyle of most PLWHIV was unsatisfactory. The booklet improved lifestyle and ART adherence in IG compared to baseline and CG.
Efficacy of a mobile phone-based intervention on health behaviors and HIV/AIDS treatment management: randomized controlled trial^(24)^/Vietnam	Assess the feasibility and effectiveness of mHealth intervention on ART adherence, self-efficacy and health behavior in PLWHIV in Vietnam.	Total: 425 IG: 238 CG: 187	Regular HIV/AIDS consultations, use of the Ecare app and one and three month follow-up.	Regular HIV/AIDS consultations. One and three month follow-up.	IG increased adherence to ART after one and three months, compared to CG. Improvement in risk behavior (smoking, drugs and alcohol) was positive in IG, but limited.
A randomized trial of a proactive cellular telephone intervention for smokers living with HIV/AIDS^(22)^/United States	Assess the effectiveness of an intervention for PLWHIV to cease smoking.	Total: 95 IG: 48 CG: 47	Standard medical advice and telephone intervention, with eight proactive face-to-face counseling sessions.	Standard counseling for smoking cessation.	IG was 3.6 times more likely to quit smoking compared to CG (p = 0.0059). Abstinence rates were 10.3% in CG and 36.8% in IG.
Effect of a WeChat-Based Intervention (Run4Love) on depressive symptoms among people living with HIV in China: randomized controlled trial^(25)^/China	Assess the effectiveness of a WeChat-based intervention (Run4Love) using a randomized clinical trial with 300 PLWHIV with depression in China.	Total: 300 PLWHIV with depression IG: 150 CG: 150	They received the Run4Love program with a course on stress management and coping, short articles on self-care and a physical activity promotion program, with established goals and personalized feedback.	They received a document on nutrition and routine care for PLWHIV on WeChat.	IG had reduced depression severity (23.9 to 17.7 versus 24.3 to 23.8), improved quality of life (77.4 to 82.6 versus 76.6 to 77.0), reduced stress (20.0 to 15.7 versus 20.7 to 18.9), and improved coping (18.4 to 20.7 versus 18.3 to 17.8) compared to CG.

Notes: ART – antiretroviral therapy; PLWHIV – people living with HIV; IG – intervention group; CG – control group; CI – confidence interval.

The studies assessed different technologies for promoting healthy lifestyles in PLWHIV. The most commonly used technologies were mobile applications^(21,23,24,25)^, printed booklets^(3,11)^, motivational interviews^(20)^, and telephone interventions^(20,22)^. The technologies were used to promote adherence to ART^(11,20,24)^, mental health, with stress, fatigue, and depression management practices^(21,25)^, encouragement of smoking cessation and reduction^(22,24)^, and reduction of risk behaviors, such as alcohol and drug use^(24)^. Studies that encouraged self-care^(3,11,23,25)^ and aspects of a healthy lifestyle, such as nutrition, physical exercise, preventive behavior, relationships and stress management, were also identified^(3,11,25)^.

All technologies were effective in improving PLWHIV’s lifestyle^(3,11,20,21,22,23,24,25)^. As for the classification of technology types^(13)^, the studies included soft-hard^(3,11,20)^ and hard^(20,21,22,23,24,25)^ technologies. Soft-hard technologies involved educational booklets to promote a healthy lifestyle, with guidance on body weight control, healthy eating, physical exercise, control/cessation of smoking and use of alcohol/other drugs, stress control, and adherence to ART^(3,11)^, in addition to face-to-face motivational interviews for adherence to ART^(20)^. Hard technologies were mobile applications^(21,23,24,25)^ and telephone counseling^(20,22)^. Mobile applications aimed to improve stress management^(21,25)^ and fatigue^(21)^, adherence to ART^(23,24)^, promotion of self-care^(23,25)^, change in risk behaviors related to smoking, alcohol and drug use^(24)^, promotion of physical activity, and reduction of depression^(25)^. Telephone interventions promoted adherence to ART through motivational interviewing^(20)^ and smoking cessation^(22)^.

In the assessment of the risk of bias, according to RoB 2.0^(18)^, it was found that the eight studies had some risk of bias, which is shown in Table 3.

**Table 3:** Assessment of risk of bias of studies according to the Risk-of-Bias Tool For Randomized Trials^(18)^ – Fortaleza, CE, Brazil, 2024.

Risk of study bias	Articles assessed							
	(20)	(21)	(3)	(23)	(11)	(24)	(22)	(25)
1. Bias arising from the randomization process	+	−	+	+	+	+	+	?
2. Bias due to deviations from intended interventions	+	+	?	+	?	?	+	?
3. Bias due to missing outcome data	+	+	+	+	?	+	+	+
4. Bias in measurement of the outcome	?	+	+	?	+	+	?	+
5. Bias in selection of the reported result	+	+	+	−	+	+	+	+
Other biases	+	+	+	+	+	+	+	+

Legend: (+) low risk; (-) high risk; (?) some concern.

Table 4 describes in detail the biases of each of the articles analyzed. The biases related to the methodological aspects of studies are particularly noteworthy.

**Table 4:** Description of study biases according to the Risk-of-Bias Tool For Randomized Trials(18) – Fortaleza, CE, Brazil, 2024.

Study authorship	Study biases according to Risk-of-Bias Tool For Randomized Trials(18)
Dilorio et al., 2008^(20)^	The majority of the sample consisted of low-income African-American men, which prevents generalization of results. Patients without low adherence to ART were included. Difficulty in adhering to the pill counting technology, which was a device attached to the bottle cap.
Barroso et al., 2020^(21)^	Significant differences between groups, with a predominance of men and people with higher education in CG. There were three people in illicit drug recovery programs in IG and none in CG.
Lima et al., 2022^(3)^	A greater number of unemployed people in CG. Part of the study took place during the COVID-19 pandemic in 2020, which may interfere with the results due to social isolation.
Schnall et al., 2018^(23)^	The researchers did not specify the components, the organization of the study team, or whether the evaluators knew the group that received the intervention.
Lima et al., 2023^(11)^	A greater number of unemployed people were in CG. Part of the study took place during the COVID-19 pandemic in 2020, which may interfere with the results due to the social isolation of participants.
Tran et al., 2017^(24)^	There was a difference in the number of participants in groups, which was lower in CG.
Vidrine et al., 2006^(22)^	They did not report whether the outcome evaluators were aware of the intervention applied, or whether there was a script or standard operating procedure for applying the interventions.
Guo et al., 2020^(25)^	There was no blinding of the data collection team or intervention participants. It was not reported whether the application of the quality of life or depression assessment instruments in the following months occurred online or in person. The number of women in the study was not addressed, only men, homosexuals or bisexuals.

Notes: ART – antiretroviral therapy; CG – control group; IG – intervention group; COVID-19 – coronavirus-19.

In the systematic review assessment using AMSTAR^(19)^, the classification of methodological quality was considered high, since, of the 16 questions answered, only one received a “no” answer when asking whether the authors of the review reported the sources of funding for the included studies. However, this item is considered a non-critical aspect to be assessed, so it does not compromise the quality of this systematic review.

## DISCUSSION

HIV infection has evolved from a fatal disease to a chronic condition due to ART so that those affected have a longer survival rate, exposing themselves to social, psychological, biological, cultural and spiritual conditions associated with the disease, in addition to the adverse events of long-term antiretroviral agents^(23)^. Research also notes that this population has an unhealthy lifestyle, associated with a sedentary lifestyle, inadequate diet, smoking, use of alcohol and other drugs as well as anxiety, depression and fatigue^(11,21,24)^.

This study gathered articles on existing technologies for improving PLHIV’s lifestyle. The use of mobile applications^(21,23,24,25)^, printed booklets^(3,11)^, motivational interviews and telephone interventions stood out^(20,22)^. The technologies were aimed at adherence to antiretroviral agents, stress and fatigue management, physical exercise, smoking discouragement, and other aspects to promote a healthy lifestyle.

Studies with mobile applications, which are designated as hard technologies, predominated^(13,21,23,24,25)^. Mobile applications stand out for their ease of access at any time or place, which favors adherence in the health area^(25)^. However, one of the difficulties is that most applications require a cell phone and internet to access the content, which can be a problem for economically vulnerable populations^(25,26)^. Applications are part of mobile health technologies (mHealth) and allow interventions through devices, being convenient for health education^(27)^. Although they have the potential to improve the lifestyle of people with HIV, many have not had their clinical efficacy assessed, and studies with this objective are needed^(23)^.

The Cognitive Behavioral Stress Management (CBSM) application was an educational intervention for stress management, interpersonal skills, relaxation, problem-solving and coping strategies^(21)^. eCARE aimed to reduce risk behaviors in PLHIV, such as smoking, alcohol and other drug use^(24)^. Applications in the context of mHealth can help PLHIV who live far from health institutions to have access to information that would not be readily available, in addition to facilitating communication with minority groups^(21,23,25)^.

A study conducted in China incorporated CBSM into a social network to provide long-term multiple follow-up. It significantly reduced the severity of depression, improved quality of life, had good viability and high levels of user satisfaction^(25)^. However, a disadvantage observed in the use of applications is the dependence of patients on managing their health status and treatment solely through the application, requiring studies to assess and propose solutions to this issue^(24)^.

Given the disadvantages mentioned in the use of mobile applications, it is clear that health technologies that do not use telephones or the Internet are also necessary, such as booklets, which can be printed and distributed, as discussed in two studies in this review^(3,11)^. This type of technology can be delivered by healthcare professionals to PLHIV at the time of consultation or health education, but the difficulty encountered in achieving the desired effect is the need for users to know how to read and interpret the texts^(3,11)^.

It is important to note that it is necessary to understand the impact of these technologies on patients’ lives, as well as the degree of importance they have for self-care, since not all individuals are ready to receive certain technologies. For instance, the fact that booklets or access to applications are distributed, and a patient does not know how to read or does not have the technology or device necessary to access the content, can generate embarrassing situations that even involve ethical aspects. On the other hand, technologies must prepare patients for autonomy and independence, through the practice of self-care. In view of this, technologies must be designed with a view to greater inclusion and access for different types of users, such as the use of adapted technologies, with the application of video and sound, in a more comprehensive way, which, in turn, depends on current public policies, democratization of education, income distribution, in addition to free access to the internet^(23)^.

The booklet entitled “*Minha Cartilha de Motivação para Mudança! Práticas para Promoção do Estilo de Vida Saudável*” was a validated technology that showed improvements in knowledge, attitude and practice about lifestyle in PLHIV in the two, four and six months of intervention, compared to the control group^(3,11)^. The booklets are a low-cost technology, easy to handle, understand and transport, that have a positive impact and generate behavior changes^(28)^.

Technological interventions in health are important for PLHIV and represent continued and additional care from healthcare professionals that goes beyond the office^(11)^. Despite the variety of educational materials available, the booklets have an objective approach to information and self-care practices, with easy-to-understand language, promoting positive behaviors in PLHIV and improving the bond with the health team^(11,29)^. Several topics have already been covered in booklets for PLHIV, such as sexual and reproductive health for serodiscordant couples^(30)^, prevention of vertical transmission of HIV^(28)^, as well as general topics on health promotion, such as tackling stigma and discrimination, the rights of PLHIV and physical health^(29)^.

Two other studies used motivational interviewing and telehealth, both through telephone calls, which are considered hard technologies^(13,20,22)^. Although mobile applications have gained ground in recent years, interventions via phone calls are a good strategy for reaching people without internet access or who do not know how to use the applications. However, time and space are required to receive the calls, as some PLHIV suffer from stigma and do not share their diagnosis, so they cannot answer calls in public, requiring a private environment^(31)^.

Motivational interviewing promotes collaboration between healthcare professionals and patients and focuses on motivation for change, respect for autonomy, empathy and professional commitment^(32)^. It was also effective in another study with PLHIV, in which there was a 34% reduction in alcohol consumption, with an impact on health and improved lifestyle^(33)^. This technology was also applied to reduce drug use and sexual risk, through a pilot study with 50 male couples, resulting in a reduction in unprotected anal sex and the use of illicit drugs^(34)^.

Despite the high number of studies identified on the subject, when the materials were read in depth, research on the construction and validation of technologies predominated, without clinical trials, which limited the sample of articles in this review. Furthermore, the impossibility of performing a meta-analysis based on selected studies was considered a limitation of this systematic review, because the methodological characteristics of clinical trials made it impossible to calculate the summary measures.

For future studies, it is suggested that the technologies be applied in randomized clinical trials to assess the effectiveness of interventions in the long term, and that these studies strictly follow the CONsolidated Standards Of Reporting Trials (CONSORT) in order to reduce bias. Moreover, it is important to apply these technologies with PLHIV from different geographic regions, to diversify the populations analyzed, as well as to assess the costs and feasibility of their implementation in healthcare services, populations or communities.

## CONCLUSION

The technologies used to promote a healthy lifestyle among PLWHIV have proven effective in terms of adherence to ART, stress management, self-care promotion, and reduction in smoking and risk behaviors, with emphasis on the use of apps, booklets, and phone calls. The implementation of technologies in the health field is on the rise, making it necessary to expand these resources among PLWHIV, a population with needs focused not only on infection, but also on promoting a healthy lifestyle in order to prevent other chronic conditions resulting from aging, HIV infection itself, and long-term use of ART.
