# Supplementary material for: Technologies for healthy lifestyle in people with HIV: a systematic review
Source: Rev Esc Enferm USP. 2025 May 9;59:e20240396. doi: 10.1590/1980-220X-REEUSP-2024-0396en (PMC12064087; doi:10.1590/1980-220X-REEUSP-2024-0396en)
Supplement: Translation

# Translation: Tecnologias para estilo de vida saudável em pessoas com HIV: revisão sistemática

**DOI:** 10.1590/1980-220X-REEUSP-2024-0396pt

**Authors:** Gilmara Holanda da Cunha, Yrene Esperanza Urbina Rojas, Maiara Bezerra Dantas, Maria Elisa Curado Gomes, Larissa Rodrigues Siqueira, Marina Soares Monteiro Fontenele

**Affiliations:** 1 Universidade Federal do Ceará, Fortaleza, CE, Brasil.; 2 Universidad Nacional de Tumbes, Tumbes, Peru.

**Keywords:** HIV, Síndrome da Imunodeficiência Adquirida, Estilo de Vida Saudável, Tecnologia Educacional, Enfermagem

## Abstract

**Objetivo:**

Analisar os ensaios clínicos que avaliaram a eficácia de tecnologias para estilo de vida saudável em pessoas com HIV.

**Método:**

Revisão sistemática, realizada em cinco bases de dados, com associação de descritores controlados. Foram incluídos artigos de ensaios clínicos randomizados controlados, completos e disponíveis eletronicamente, sem restrição de idioma ou data, envolvendo tecnologias para estilo de vida saudável em pessoas com HIV maiores de 18 anos. Foram excluídos os estudos com crianças, adolescentes, gestantes e artigos repetidos. Para avaliar os vieses e a qualidade da revisão, utilizaram-se o *Risk-of-Bias Tool For Randomized Trials* e o *Assessment of Multiple Systematic Reviews*.

**Resultados:**

Foram identificados 2.933 artigos e oito foram selecionados. As tecnologias foram aplicativos móveis, cartilhas, entrevista motivacional e intervenções por telefone, com foco no autocuidado, adesão antirretroviral, gerenciamento de estresse, fadiga e depressão, estímulo redução do tabagismo, e uso de álcool e drogas.

**Conclusão:**

As tecnologias foram classificadas como leve-duras e duras, e melhoraram o estilo de vida das pessoas com HIV. Registro no *International Prospective Register Systematic Reviews* (PROSPERO): CRD42023422772.

## INTRODUÇÃO

A terapia antirretroviral (TARV) reduziu a mortalidade por síndrome da imunodeficiência adquirida (aids) e a infecção pelo vírus da imunodeficiência humana (HIV) se tornaram uma condição crônica, com aumento da sobrevida e, concomitantemente, maior incidência de doenças não relacionadas à aids^(1)^. O controle da doença por meio dos antirretrovirais faz parte da meta 95-95-95, que tem como objetivo que, até 2030, 95% das pessoas vivendo com HIV (PVHIV) conheçam seu *status* sorológico, 95% destas estejam em tratamento antirretroviral e 95% tenham a carga viral suprimida^(2)^.

Os cuidados às PVHIV que antes estavam direcionados às infecções oportunistas foram transferidos para outras alterações de saúde que acometem a população geral^(3)^. Assim, considera-se que adotar práticas para um estilo de vida saudável, por meio da alimentação adequada, exercício físico, não uso de drogas lícitas e ilícitas, controle do estresse, e adesão aos antirretrovirais e outros fármacos, é conduta essencial para melhorar a qualidade de vida das pessoas com HIV^(4)^. Porém, constata-se uma carência no incentivo à promoção do autocuidado para o estilo de vida saudável entre esses pacientes^(5)^.

Estudos mostram que, embora as PVHIV tenham um aumento da sobrevida pela TARV, as doenças crônicas não transmissíveis (DCNTs) ocorrem com maior frequência nesse público, destacando-se a doença pulmonar obstrutiva crônica, a cardiopatia isquêmica, as doenças mentais, e as disfunções renais e hepáticas^(6,7)^. O estilo de vida é um fator para o aparecimento e manutenção das DCNT, e modificações nesse aspecto representam intervenção importante para prevenir comorbidades e enfrentar doenças^(8)^. Ferramentas de cuidado são necessárias para o aconselhamento contínuo das PVHIV, bem como intervenções de educação em saúde que incentivem a participação ativa do paciente no plano terapêutico^(9,10)^.

Diante disso, as tecnologias surgem como ferramentas facilitadoras do cuidado capazes de disseminar informações precisas e conhecimentos indispensáveis^(11)^. Estudos mostram que as tecnologias podem ajudar as PVHIV a melhorarem seu estilo de vida por meio da mudança de hábitos^(9,10)^. O avanço das tecnologias proporciona inovações na área da saúde, sensibilizando as PVHIV quanto ao autocuidado, contribuindo no acesso às informações e direcionando os que precisam de serviços de saúde^(5)^.

São consideradas tecnologias em saúde manuais, cartilhas, *folders*, *e-books*, programas educativos e *softwares* ^(12)^, que têm um papel crucial no desenvolvimento de ações preventivas do HIV e no acompanhamento dos pacientes^(12)^. As tecnologias em saúde podem ser classificadas em leves (tecnologias das relações, de produção e comunicação, que envolvem acolhimento, vínculo e escuta); leve-duras (saberes bem estruturados no processo de saúde, formulação de materiais educativos e aplicação de teorias); e duras (equipamentos tecnológicos, instrumentos, normas e s*oftwares*)^(13)^. As tecnologias podem auxiliar os enfermeiros e demais membros da equipe multiprofissional de saúde nas atividades de assistência e orientação, de forma a contribuir no autocuidado e adesão ao estilo de vida saudável pelas PVHIV^(9)^. Entretanto, antes que possam ser utilizadas pelos profissionais de saúde e pacientes, devem ter sua eficácia avaliada.

As tecnologias eficazes em saúde para a promoção do estilo de vida saudável colaboram tanto na promoção do cuidado pelos profissionais quanto na autonomia e adesão a esses cuidados pelas PVHIV. Avaliar se essas tecnologias são eficazes pode favorecer a prática baseada em evidências, visto que há vários desafios para a implementação do cuidado nessa população, tais como a dificuldade em acessar os serviços de saúde, devido ao estigma decorrente da doença e medo da divulgação do *status* sorológico^(1,11)^, baixo letramento funcional em saúde^(3)^, além da baixa escolaridade e renda^(2,3,9,11)^. Todos esses aspectos podem impactar negativamente a assistência em saúde e o estilo de vida.

Diante do exposto, este estudo foi orientado pela seguinte questão de pesquisa: qual a eficácia das tecnologias utilizadas para promover o estilo de vida saudável em PVHIV? Seu objetivo foi analisar os ensaios clínicos que avaliaram a eficácia de tecnologias para o estilo de vida saudável em pessoas com HIV.

## MÉTODO

### Tipo de Estudo

Trata-se de revisão sistemática, com abordagem quantitativa, descritiva e sem metanálise, realizada em seis etapas: 1. Elaboração da pergunta norteadora; 2. Formação de um protocolo; 3. Geração de uma lista de estudos relevantes; 4. Seleção dos estudos para análise; 5. Avaliação da qualidade dos estudos e extração de dados; 6. Síntese e escrita do manuscrito^(14)^.

Foram seguidas as recomendações do *Preferred Reporting Items for Systematic Reviews and Meta-Analyses* (PRISMA)^(15)^, e a revisão sistemática foi cadastrada no *International Prospective Register of Systematic Reviews* (PROSPERO) em 2023, sob registro N° CRD42023422772.

### Local e Período do Estudo

O levantamento dos artigos nas bases de dados ocorreu no período de outubro de 2023 a janeiro de 2024 por pesquisadores localizados em Fortaleza, Ceará, Brasil, e em Tumbes, Peru.

### Critérios de Elegibilidade

A questão da pesquisa foi feita de acordo com a estratégia PICO, acrônimo para Paciente (pessoas com HIV), Intervenção (eficácia de tecnologias para o estilo de vida saudável), Comparação (não aplicada no estudo, pois o objetivo não foi comparar intervenções) e *Outcome* ou Desfecho (melhora do estilo de vida). A revisão sistemática teve como pergunta norteadora: qual a eficácia das tecnologias utilizadas para o estilo de vida saudável em PVHIV?

Os critérios de inclusão foram estudos do tipo ensaio clínico randomizado, classificados com nível de evidência II, que são os ensaios clínicos randomizados e controlados^(16)^, além de artigos completos disponíveis eletronicamente, sem restrição de idioma ou data de publicação, envolvendo as tecnologias para o estilo de vida saudável em PVHIV maiores de 18 anos. Os critérios de exclusão foram estudos com crianças, adolescentes e gestantes, além de artigos repetidos, os quais foram contabilizados uma única vez.

### Fontes de Informação e Estratégias de Busca nas Bases de Dados

Os artigos foram selecionados em cinco bases de dados: *Medical Literature Analysis and Retrieval System Online* (MEDLINE); Scopus; Embase; *Web of Science*; e Literatura Latino-Americana e do Caribe em Ciências da Saúde (LILACS). A escolha das bases ocorreu a partir da pergunta norteadora, tipos de estudos desejados e visibilidade na área da saúde.

Foram utilizados os descritores “*HIV”*, “*HIV Infections”,* “*Acquired Immunodeficiency Syndrome”, “Technology”*, “*Technologies”*, *“Life Style”* e *“Healthy Lifestyle”,* todos provenientes dos Descritores em Ciências da Saúde (DeCS) da Biblioteca Virtual em Saúde e do *Medical Subject Headings* (MeSH) da *National Library of Medicine.* Os descritores foram readequados conforme o local de busca: inseridos no idioma inglês, selecionando os operadores booleanos *AND* e *OR* no MEDLINE, Scopus e *Web of Science*; inseridos nos idiomas português, inglês e espanhol, e o operador booleano *AND* na LILACS; inseridos no idioma inglês, selecionando-se a opção com booleano *AND* na Embase. As estratégias de busca e o número de artigos encontrados estão descritos na Tabela 1.

**Tabela 1:** Estratégias de busca dos artigos nas bases de dados – Fortaleza, CE, Brasil, 2024.

Bases de dados	Cruzamentos nas bases de dados	Número de artigos
MEDLINE	(“Acquired Immunodeficiency Syndrome”[Mesh] OR “HIV”[Mesh]) AND “Technology”[Mesh]	1.371
Scopus	(Technologies OR Technology) AND Lifestyle AND HIV	53
Embase	(‘Human Immunodeficiency Virus’/exp OR ‘Human Immunodeficiency Virus’ OR ‘Acquired Immune Deficiency Syndrome’/exp OR ‘Acquired Immune Deficiency Syndrome’) AND (‘Technology’/exp OR Technology) AND (‘Lifestyle’/exp OR Lifestyle)	218
Web of Science	(“HIV Infections” OR “Acquired Immunodeficiency Syndrome”) AND (Technology OR Technologies)	841
LILACS	(HIV) AND (Technology) AND (“Healthy Lifestyle”) OR (“Life Style”)	196

### Processo de Extração e Análise das Informações dos Estudos Selecionados

A seleção e análise dos artigos foram realizadas no período de fevereiro a maio de 2024, por dois revisores independentes, e um terceiro foi utilizado para definir os casos de discordância entre os demais. Após as buscas nas bases de dados eletrônicas, os artigos foram exportados para o Rayyan, ferramenta *online* para construção de revisões sistemáticas^(17)^. Inicialmente, removeram-se os estudos duplicados e, em seguida, realizou-se a leitura de todos os títulos e resumos para identificar os estudos relevantes, considerando-se os critérios de inclusão e exclusão. Foram lidos na íntegra os artigos em que esses critérios não estavam claros. Posteriormente, ocorreu a avaliação da elegibilidade pela leitura integral dos estudos selecionados. Os dados foram extraídos dos artigos e organizados em fichas clínicas com as informações de título, autoria, ano de publicação, país de realização do estudo, objetivos, amostra, grupos intervenção e controle, desfechos, e vieses.

### Avaliação dos Vieses dos Estudos

Para avaliação dos vieses das pesquisas, utilizou-se a ferramenta *Risk-of-Bias Tool For Randomized Trials* (RoB 2.0)^(18)^. Para avaliar a qualidade da revisão sistemática, utilizou-se o *Assessment of Multiple Systematic Reviews* (AMSTAR)^(19)^.

Os oito artigos que atenderam à questão de pesquisa foram analisados por abordagem organizada para ponderar o rigor e as características dos estudos, observando-se desenvolvimento metodológico, intervenção, amostra, resultados, conclusão e possíveis vieses da pesquisa. A avaliação do risco de viés dos estudos foi feita pela ferramenta RoB 2.0 em cinco domínios: 1. Viés decorrente do processo de randomização; 2. Viés decorrente do desvio da intervenção pretendida; 3. Viés decorrente de dados dos resultados ausentes; 4. Viés decorrente da medição dos resultados; e 5. Viés decorrente da seleção dos resultados reportados. Cada domínio foi avaliado como baixo risco de viés, alto risco de viés ou alguma preocupação^(18)^.

O AMSTAR avaliou a qualidade da revisão sistemática. Trata-se de instrumento com 16 itens, que correspondem aos requisitos mínimos de uma revisão sistemática^(19)^: 1. As questões de pesquisa e critérios de inclusão para revisão incluíram os componentes do PICO? 2. O relatório da revisão continha uma declaração explícita de que os métodos de revisão foram estabelecidos antes da revisão e o relatório justificava quaisquer desvios significativos do protocolo? 3. Os autores da revisão explicaram a seleção dos desenhos de estudo para inclusão na revisão? 4. Os autores da revisão utilizaram uma estratégia abrangente de pesquisa bibliográfica? 5. Os revisores realizaram a seleção dos estudos em duplicata? 6. Os revisores realizaram a extração de dados em duplicata? 7. Os autores da revisão forneceram uma lista de estudos excluídos e justificaram as exclusões? 8. Os autores da revisão descreveram os estudos incluídos com detalhes adequados? 9. Os autores da revisão utilizaram uma técnica satisfatória para avaliar o risco de viés em estudos individuais incluídos na revisão? 10. Os autores da revisão informaram as fontes de financiamento dos estudos incluídos na revisão? 11. Se foi realizada metanálise, os autores da revisão utilizaram métodos apropriados para combinação estatística dos resultados? 12. Se a metanálise foi realizada, os autores da revisão avaliaram o impacto potencial do risco de viés em estudos individuais sobre os resultados da metanálise ou de outra síntese de evidências? 13. Os autores da revisão levaram em conta o risco de viés nos estudos primários ao interpretar/discutir os resultados da revisão? 14. Os autores da revisão forneceram uma explicação satisfatória e discussão sobre qualquer heterogeneidade observada nos resultados da revisão? 15. Se realizaram uma síntese quantitativa, os autores da revisão investigaram adequadamente o viés de publicação (viés de pequenos estudos) e discutiram seu provável impacto nos resultados da revisão? 16. Os autores da revisão relataram fontes potenciais de conflito de interesses e financiamentos que receberam para conduzir a revisão?

Todos os itens são respondidos com “sim” para os resultados positivos, “não”, nos casos em que não havia informações disponíveis ou o avaliador julgou que não podia optar pelo benefício da dúvida, ou “parcialmente sim”, quando foi considerado válido indicar adesão parcial ao domínio. Sete dos 16 itens são críticos (1, 4, 7, 9, 11, 13 e 15). Ao final da avaliação, a revisão é classificada como criticamente baixa (mais de uma falha crítica), baixa (uma falha crítica), moderada (mais de uma falha não crítica) e alta (nenhuma falha ou uma falha não crítica)^(19)^. O AMSTAR foi aplicado de forma independente por dois avaliadores, e as diferenças nas avaliações foram discutidas e acordadas por consenso. Ao final, os achados dos artigos foram discutidos segundo a literatura científica.

### Aspectos Éticos

Quanto aos aspectos éticos, respeitaram-se os escritos dos artigos e os direitos autorais, sem modificação do conteúdo identificado, em benefício deste estudo proposto pelos autores.

## RESULTADOS

Foram identificados 2.679 artigos e, após as análises, um total de oito compuseram o estudo. A Figura 1 mostra o fluxograma com o quantitativo de artigos selecionados.

**Figura 1:** Fluxograma de seleção dos estudos, adaptado do *Preferred Reporting Items for Systematic Reviews and Meta-Analyses* – Fortaleza, CE, Brasil, 2024.

O ano de publicação dos artigos variou entre 2006 e 2023, sendo três realizados nos Estados Unidos, dois, no Brasil, um, no Canadá, um, no Vietnã, e um, na China^(3,11,20,21,22,23,24,25)^. A caracterização dos artigos quanto ao título, país de realização do estudo, objetivos, amostra, grupos e desfechos encontra-se na Tabela 2.

**Tabela 2:** Estudos sobre tecnologias para estilo de vida saudável em pessoas com HIV segundo título, país de realização do estudo, objetivos, amostra, grupos e desfechos Fortaleza, CE, Brasil, 2024.

Título/país de realização do estudo	Objetivos	Amostra	Grupo intervenção	Grupo controle	Desfechos
Using motivational interviewing to promote adherence to antiretroviral medications: a randomized controlled study(20)/Estados Unidos	Avaliar a entrevista motivacional para melhorar a adesão à TARV em PVHIV.	Total: 247 GI: 125 GC: 122	Entrevista motivacional presencial, aplicada por enfermeiro em cinco sessões, com duração de 20-90 minutos, por três meses presenciais e por telefone.	Cuidado padrão da instituição.	GI foi superior ao GC na adesão à TARV após seis meses do início da intervenção (p < 0,001).
A feasibility study to develop and test a cognitive behavioral stress management mobile health application for HIV-related fatigue(21)/Estados Unidos	Determinar a viabilidade e aceitabilidade do aplicativo CBSM *mHealth* em smartphone, para enfrentar e gerenciar o estresse e fadiga em PVHIV.	Total: 30 GI: 15 GC: 15	Baixava e utilizava o aplicativo CBSM *mHealth* em seus smartphones, e foram solicitados a concluir um módulo por semana.	Baixava o aplicativo *Lifesum*, que é um aplicativo genérico sobre estilo de vida saudável, e utilizava uma vez na semana.	GI: redução da fadiga superior ao GC: (diferença média: 1,6; IC 95%: 0,3-2,8). GI: diminuição superior na depressão até a 5ª semana (diferença média: 9,7; IC 95%: 1,1-18,4).
Knowledge, attitude and practice of people with HIV regarding a healthy lifestyle: clinical trial(3)/Brasil	Avaliar a efetividade de uma cartilha sobre o conhecimento, atitude e prática para estilo de vida saudável em PVHIV.	Total: 144 GI: 70 GC: 74	Recebeu a cartilha em consultório, fez a leitura completa e levou-a para casa para ler a cada 15 dias por seis meses.	Cuidado habitual do serviço.	A cartilha educativa melhorou o conhecimento, atitude e prática sobre o estilo de vida saudável nas PVHIV.
Mobile health technology for improving symptom management in low income persons living with HIV(23)/Canadá	Examinar o impacto de um aplicativo de *mHealth* (*mVip*) com estratégias de autocuidado para sintomas de PVHIV.	Total: 76 GI: 40 GC: 40	Faziam login no *mVip* ao menos uma vez por semana para avaliação de sintomas e receber orientações de autocuidado.	Baixaram o *mVIP*, mas não receberam estratégias de autocuidado.	GI: melhora em 12 dos 13 sintomas da infecção pelo HIV e na adesão à TARV (p = 0,017), em comparação ao GC.
Booklet for healthy lifestyle in people with HIV: a clinical trial(11)/Brasil	Avaliar a efetividade de uma cartilha educativa para promoção do estilo de vida saudável e adesão à TARV em PVHIV.	Total: 174 GI: 70 GC: 74	Consulta médica de rotina, leitura da cartilha no ambulatório, levando a cartilha para casa para leitura quinzenal por seis meses.	Consulta médica de rotina.	Na avaliação basal, o estilo de vida da maioria das PVHIV era insatisfatório. A cartilha melhorou o estilo de vida e adesão à TARV no GI, comparado ao basal e ao GC.
Efficacy of a mobile phone-based intervention on health behaviors and HIV/AIDS treatment management: randomized controlled trial(24)/Vietnã	Avaliar a viabilidade e eficácia da intervenção de *mHealth* na adesão à TARV, autoeficácia e comportamento de saúde em PVHIV no Vietnã.	Total: 425 GI: 238 GC: 187	Consultas regulares para HIV/aids, uso do aplicativo Ecare e acompanhamento de um e três meses.	Consultas regulares para HIV/aids. Acompanhamento de um e três meses.	O GI aumentou a adesão à TARV após um e três meses, comparado ao GC. A melhora do comportamento de risco (tabagismo, drogas e álcool) foi positiva no GI, mas limitada.
A randomized trial of a proactive cellular telephone intervention for smokers living with HIV/AIDS(22)/Estados Unidos	Avaliar a eficácia de uma intervenção para PVHIV cessarem o tabagismo.	Total: 95 GI: 48 GC: 47	Aconselhamento médico padrão e intervenção por telefone, com oito sessões proativas de aconselhamento presencial.	Aconselhamento padrão para cessação do tabagismo.	GI teve 3,6 vezes mais chances de parar de fumar, comparado ao GC (p = 0,0059). Taxas de abstinência foram de 10,3% no GC e 36,8% no GI.
Effect of a WeChat-Based Intervention (Run4Love) on depressive symptoms among people living with HIV in China: randomized controlled trial(25)/China	Avaliar a eficácia de uma intervenção baseada no *WeChat* (*Run4Love*), com um ensaio clínico randomizado com 300 PVHIV com depressão na China.	Total: 300 PVHIV com depressão GI: 150 GC: 150	Receberam o programa *Run4Love* com um curso sobre gestão e enfrentamento da redução do estresse, artigos curtos sobre autocuidado e programa de promoção de atividade física, com metas estabelecidas e *feedback* personalizado.	Receberam no *WeChat* um documento sobre nutrição e cuidados habituais para PVHIV.	GI teve redução da gravidade da depressão (23,9 para 17,7 *versus* 24,3 para 23,8), melhora da qualidade de vida (77,4 para 82,6 *versus* 76,6 para 77,0), redução do estresse (20,0 para 15,7 *versus* 20,7 para 18,9) e melhora do enfrentamento (18,4 para 20,7 *versus* 18,3 para 17,8), comparado ao GC.

Notas: TARV – terapia antirretroviral; PVHIV – pessoas vivendo com HIV; GI – grupo intervenção; GC – grupo controle; IC – Intervalo de Confiança.

Os estudos avaliaram diferentes tecnologias para promoção do estilo de vida saudável em PVHIV. As tecnologias mais utilizadas foram os aplicativos móveis^(21,23,24,25)^, cartilhas impressas^(3,11)^, entrevista motivacional^(20)^ e intervenções por telefone^(20,22)^. As tecnologias foram utilizadas para promover adesão à TARV^(11,20,24)^, saúde mental, com práticas de gerenciamento de estresse, fadiga e depressão^(21,25)^, estímulo à cessação e redução do tabagismo^(22,24)^, e redução de comportamentos de risco, como o uso de álcool e drogas^(24)^. Também foram identificados estudos que estimulavam o autocuidado^(3,11,23,25)^ e aspectos do estilo de vida saudável, como nutrição, prática de exercício físico, comportamento preventivo, relacionamentos e controle do estresse^(3,11,25)^.

Todas as tecnologias tiveram eficácia para melhorar o estilo de vida das PVHIV^(3,11,20–25)^. Quanto à classificação dos tipos de tecnologia^(13)^, os estudos contemplaram as tecnologias leve- duras^(3,11,20)^ e duras^(20–25)^. As tecnologias leves-duras envolveram cartilhas educativas para promoção de estilo de vida saudável, com orientações sobre controle do peso corporal, alimentação saudável, prática de exercício físico, controle/cessação do tabagismo e uso do álcool/outras drogas, controle do estresse, e aderência à TARV^(3,11)^, além da entrevista motivacional presencial para adesão à TARV^(20)^. As tecnologias duras foram os aplicativos móveis^(21,23,24,25)^ e aconselhamento por telefone^(20,22)^. Os aplicativos móveis visavam melhorar o gerenciamento do estresse^(21,25)^ e fadiga^(21)^, adesão à TARV^(23,24)^, promoção do autocuidado^(23,25)^, mudança de comportamento de risco no tabagismo, uso de álcool e drogas^(24)^, promoção de atividade física, e redução da depressão^(25)^. As intervenções por ligação telefônica promoveram a adesão à TARV por meio da entrevista motivacional^(20)^ e cessação do tabagismo^(22)^.

Na avaliação do risco de viés, segundo o RoB 2.0^(18)^, constatou-se que os oito estudos possuíam algum risco de viés, o que é mostrado na Tabela 3.

**Tabela 3:** Avaliação do risco de viés dos estudos segundo o Risk-of-Bias Tool For Randomized Trials(18) – Fortaleza, CE, Brasil, 2024.

Risco de viés dos estudos	Artigos avaliados							
	(20)	(21)	(3)	(23)	(11)	(24)	(22)	(25)
1. Viés decorrente do processo de randomização	+	−	+	+	+	+	+	?
2. Viés decorrente do desvio da intervenção pretendida	+	+	?	+	?	?	+	?
3. Viés decorrente de dados dos resultados ausentes	+	+	+	+	?	+	+	+
4. Viés decorrente da medição dos resultados	?	+	+	?	+	+	?	+
5. Viés decorrente da seleção dos resultados reportados	+	+	+	−	+	+	+	+
Outros vieses	+	+	+	+	+	+	+	+

Legenda: (+) baixo risco; (−) alto risco; (?) alguma preocupação.

Na Tabela 4, são descritos de maneira detalhada os vieses de cada um dos artigos analisados. Ressaltam-se, sobretudo, os vieses relacionados aos aspectos metodológicos dos estudos.

**Tabela 4:** Descrição dos vieses dos estudos segundo o Risk-of-Bias Tool For Randomized Trials^(18)^ – Fortaleza, CE, Brasil, 2024.

Autoria dos estudos	Vieses dos estudos segundo o *Risk-of-Bias Tool For Randomized Trials*(18)
Dilorio et al., 2008^(20)^	Maioria da amostra foi de homens afro-americanos de baixa renda, o que impede de generalizar os resultados. Foram incluídos pacientes sem baixa adesão à TARV. Dificuldade em aderir à tecnologia de contagem de comprimidos, que era um dispositivo acoplado à tampa do frasco.
Barroso et al., 2020^(21)^	Diferenças significantes entre os grupos, com predomínio de homens e pessoas com nível educacional superior no GC. Havia três pessoas em programas de recuperação contra drogas ilícitas no GI e nenhuma no GC.
Lima et al., 2022^(3)^	Maior número dos desempregados no GC. Parte do estudo ocorreu na pandemia de COVID-19, em 2020, o que pode interferir nos resultados, devido ao isolamento social.
Schnall et al., 2018^(23)^	Os pesquisadores não especificaram os componentes, a organização da equipe do estudo e se os avaliadores conheciam o grupo que recebeu a intervenção.
Lima et al., 2023^(11)^	Maior número de desempregados estavam no GC. Parte do estudo aconteceu na pandemia de COVID-19, em 2020, o que pode interferir nos resultados, devido ao isolamento social dos participantes.
Tran et al., 2017^(24)^	Houve diferença na quantidade de participantes nos grupos, que foi menor no GC.
Vidrine et al., 2006^(22)^	Não informaram se os avaliadores dos resultados tinham conhecimento sobre a intervenção aplicada, se havia roteiro ou procedimento operacional padrão para aplicar as intervenções.
Guo et al., 2020^(25)^	Não houve cegamento da equipe de coleta de dados e dos participantes da intervenção. Não foi relatado se a aplicação dos instrumentos de avaliação da qualidade de vida ou depressão nos meses posteriores ocorreu de forma online ou presencial. Não foi abordado o quantitativo de mulheres no estudo, apenas homens, homossexuais ou bissexuais.

Notas: TARV – terapia antirretroviral; GC – grupo controle; GI – grupo intervenção; COVID-19 – coronavírus-19.

Na avaliação da revisão sistemática por meio do AMSTAR^(19)^, a classificação da qualidade metodológica foi considerada alta, visto que, das 16 perguntas respondidas, apenas uma obteve “não” como resposta ao questionar se os autores da revisão informaram as fontes de financiamento dos estudos incluídos. No entanto, este item é considerado um aspecto não crítico a ser avaliado, de forma que não compromete a qualidade da presente revisão sistemática.

## DISCUSSÃO

A infecção pelo HIV evoluiu de uma doença fatal para uma condição crônica devido à TARV, de forma que os acometidos têm maior sobrevida, expondo-se a condições sociais, psicológicas, biológicas, culturais e espirituais associadas à doença, além dos eventos adversos dos antirretrovirais a longo prazo^(23)^. Pesquisas também observam que essa população possui um estilo de vida não saudável, associado ao sedentarismo, alimentação inadequada, tabagismo, uso de álcool e outras drogas, além de ansiedade, depressão e fadiga^(11,21,24)^.

Este estudo reuniu artigos sobre as tecnologias existentes para melhoria do estilo de vida em PVHIV. Destacou-se a utilização dos aplicativos móveis^(21,23,24,25)^, cartilhas impressas^(3,11)^, entrevista motivacional e intervenções telefônicas^(20,22)^. As tecnologias foram direcionadas à adesão aos antirretrovirais, gerenciamento do estresse e fadiga, prática de exercício físico, desencorajamento do tabagismo, além de outros aspectos para promover o estilo de vida saudável.

Predominaram estudos com aplicativos móveis, os quais são designados tecnologias duras^(13,21,23,24,25)^. Os aplicativos móveis destacam-se pela facilidade de acesso a qualquer hora ou lugar, o que favorece a adesão na área da saúde^(25)^. No entanto, uma das dificuldades é que a maioria dos aplicativos necessita de aparelho celular e internet para acessar os conteúdos, o que pode ser um problema para as populações com vulnerabilidade econômica^(25,26)^. Os aplicativos inserem-se nas tecnologias móveis em saúde (*mHealth*) e permitem intervenções por dispositivos, sendo convenientes para a educação em saúde^(27)^. Embora tenham potencial para melhorar o estilo de vida das pessoas com HIV, muitos não têm sua eficácia clínica avaliada, sendo necessários estudos com esse objetivo^(23)^.

O aplicativo *Cognitive Behavioral Stress Management* (CBSM) foi uma intervenção educativa para gerenciamento do estresse, habilidades interpessoais, relaxamento, resolução de problemas e estratégias de enfrentamento^(21)^. Já o eCARE visou à diminuição dos comportamentos de risco em PVHIV, tais como a redução do tabagismo, do uso de álcool e outras drogas^(24)^. Aplicativos no contexto da *mHealth* podem ajudar as PVHIV que residem longe das instituições de saúde a terem acesso às informações que não estariam prontamente disponíveis, além de facilitar a comunicação com os grupos minoritários^(21,23,25)^.

Estudo realizado na China incorporou o CBSM a uma rede social para fornecer o acompanhamento múltiplo em longo prazo. Reduziu significativamente a gravidade da depressão, melhorou a qualidade de vida, teve boa viabilidade e elevado nível de satisfação dos usuários^(25)^. Porém, uma desvantagem observada no uso dos aplicativos é a dependência dos pacientes em gerenciar seu estado de saúde e tratamento apenas por meio do aplicativo, sendo necessários estudos que avaliem e proponham soluções para essa questão^(24)^.

Diante das desvantagens citadas no uso de aplicativos móveis, percebe-se que também são necessárias tecnologias em saúde que não utilizem aparelhos telefônicos ou a internet, como as cartilhas, que podem ser impressas e distribuídas, como abordado em dois estudos desta revisão^(3,11)^. Esse tipo de tecnologia pode ser entregue pelos profissionais de saúde às PVHIV no momento da consulta ou da educação em saúde, mas a dificuldade encontrada para atingir o efeito desejado é a necessidade de o usuário saber ler e interpretar os textos^(3,11)^.

Ressalta-se que é necessário saber do impacto dessas tecnologias na vida dos pacientes, bem como o grau de importância que elas têm para a condução do autocuidado, pois nem todos os indivíduos estão prontos para receberem determinadas tecnologias. Por exemplo, o fato de serem distribuídas cartilhas ou acesso a aplicativos, e algum paciente não souber ler ou não tiver a tecnologia ou dispositivo necessário para acessar o conteúdo, pode gerar situações constrangedoras que envolvem até mesmo aspectos éticos. Por outro lado, as tecnologias devem preparar o paciente para a autonomia e independência, por meio da prática do autocuidado. Diante disso, as tecnologias devem ser elaboradas visando a maior inclusão e acesso dos diferentes tipos de usuários, como o uso de tecnologias adaptadas, com aplicação de vídeo e som, de forma mais abrangente, que, por sua vez, depende das políticas públicas vigentes, democratização do ensino, distribuição de renda, além do acesso gratuito à rede de internet^(23)^.

A cartilha intitulada “Minha Cartilha de Motivação para Mudança! Práticas para Promoção do Estilo de Vida Saudável” foi uma tecnologia validada que mostrou melhora de conhecimento, atitude e prática sobre estilo de vida em PVHIV nos dois, quatro e seis meses de intervenção, em comparação ao grupo controle^(3,11)^. As cartilhas são uma tecnologia de baixo custo, fácil manuseio, entendimento e transporte que têm impacto positivo e geram mudanças de comportamento^(28)^.

As intervenções tecnológicas em saúde são importantes para as PVHIV e representam um cuidado continuado e adicional dos profissionais de saúde que vai além do consultório^(11)^. Apesar da variedade de materiais educativos disponíveis, as cartilhas têm uma abordagem objetiva de informações e práticas de autocuidado, com linguagem de fácil entendimento, favorecendo os comportamentos positivos em PVHIV e melhorando o vínculo com a equipe de saúde^(11,29)^. Diversos são os temas já abordados em cartilhas para PVHIV, tais como saúde sexual e reprodutiva para casais sorodiscordantes^(30)^, prevenção da transmissão vertical do HIV^(28)^, além de temas gerais sobre promoção da saúde, como o enfrentamento do estigma e da discriminação, direitos das PVHIV e saúde física^(29)^.

Outros dois estudos utilizaram a entrevista motivacional e o telessaúde, ambos por meio de chamadas telefônicas, sendo consideradas tecnologias duras^(13,20,22)^. Apesar de os aplicativos móveis ganharem espaço nos últimos anos, as intervenções por chamadas telefônicas são uma boa estratégia para alcançar as pessoas sem acesso à internet ou que não sabem manusear os aplicativos. Porém, são necessários tempo e local disponíveis para receber as ligações, pois algumas PVHIV sofrem com o estigma e não compartilham seu diagnóstico, de forma que não podem atender às ligações em público, necessitando de um ambiente privativo^(31)^.

A entrevista motivacional promove a colaboração dos profissionais de saúde com os pacientes e concentra-se na motivação para a mudança, respeito à autonomia, empatia e compromisso profissional^(32)^. Também foi eficaz em outro estudo com PVHIV, no qual houve a redução de 34% do consumo de álcool, com impacto na saúde e melhora do estilo de vida^(33)^. Essa tecnologia também foi aplicada para redução do uso de drogas e do risco sexual, por meio de estudo piloto com 50 casais masculinos, ocorrendo a redução do sexo anal sem preservativo e do uso de drogas ilícitas^(34)^.

Apesar do elevado número de estudos identificados sobre a temática, quando realizada a leitura aprofundada dos materiais, predominaram as pesquisas para construção e validação das tecnologias, sem a realização de ensaios clínicos, o que limitou a amostra de artigos na presente revisão. Ademais, foi considerada uma limitação desta revisão sistemática a impossibilidade de realizar uma metanálise a partir dos estudos selecionados, porque as características metodológicas dos ensaios clínicos inviabilizaram o cálculo das medidas resumo.

Para estudos futuros, sugere-se a aplicação das tecnologias em ensaios clínicos randomizados, para avaliar a eficácia das intervenções a longo prazo, e que essas pesquisas sigam rigorosamente o *CONsolidated Standards Of Reporting Trials* (CONSORT), a fim de reduzir os vieses. Além disso, é importante a aplicação dessas tecnologias com as PVHIV de diferentes regiões geográficas, para diversificar as populações analisadas, bem como avaliar os custos e a viabilidade de sua implementação nos serviços de saúde, populações ou comunidades.

## CONCLUSÃO

As tecnologias utilizadas para promoção do estilo de vida saudável em PVHIV se mostraram eficazes para adesão à TARV, gerenciamento do estresse, promoção do autocuidado, redução do tabagismo e comportamentos de risco, destacando-se o uso de aplicativos, cartilhas e ligações telefônicas. A implementação das tecnologias no âmbito da saúde está em ascensão, fazendo-se necessária a ampliação desses recursos entre as PVHIV, população com necessidades voltadas não apenas para a infecção, mas também para promoção do estilo de vida saudável, a fim de prevenir outras condições crônicas decorrentes do envelhecimento, da própria infecção pelo HIV e do uso da TARV em longo prazo.

# Translation: Tecnologías para un estilo de vida saludable en personas con VIH: revisión sistemática

**DOI:** 10.1590/1980-220X-REEUSP-2024-0396es

**Authors:** Gilmara Holanda da Cunha, Yrene Esperanza Urbina Rojas, Maiara Bezerra Dantas, Maria Elisa Curado Gomes, Larissa Rodrigues Siqueira, Marina Soares Monteiro Fontenele

**Affiliations:** 1 Universidade Federal do Ceará, Fortaleza, CE, Brasil.; 2 Universidad Nacional de Tumbes, Tumbes, Peru.

**Keywords:** VIH, Síndrome de Inmunodeficiencia Adquirida, Estilo de Vida Saludable, Tecnología Educacional, Enfermería

## Abstract

**Objetivo:**

Analizar ensayos clínicos que evaluaron la efectividad de tecnologías de estilo de vida saludable en personas con VIH.

**Método:**

Revisión sistemática, realizada en cinco bases de datos, con asociación de descriptores controlados. Se incluyeron artículos de ensayos clínicos controlados aleatorios, completos y disponibles electrónicamente, sin restricciones de idioma ni fecha, que involucran tecnologías para un estilo de vida saludable en personas con VIH mayores de 18 años. Se excluyeron estudios con niños, adolescentes, mujeres embarazadas y artículos repetidos. Para evaluar los sesgos y la calidad de las revisiones, se utilizaron el Risk-of-Bias Tool For Randomized Trials y el Assessment of Multiple Systematic Reviews.

**Resultados:**

Se identificaron 2.933 artículos y se seleccionaron ocho. Las tecnologías fueron aplicaciones móviles, folletos, entrevistas motivacionales e intervenciones telefónicas, centradas en el autocuidado, la adherencia a los antirretrovirales, el manejo del estrés, la fatiga y la depresión, fomentando la reducción del tabaquismo y el uso de alcohol y drogas.

**Conclusión:**

Las tecnologías fueron clasificadas en livianas y duras y mejoraron el estilo de vida de las personas con VIH. Inscripción en el International Prospective Register Systematic Reviews (PROSPERO): CRD42023422772.

## INTRODUCCIÓN

La terapia antirretroviral (TAR) ha reducido la mortalidad por síndrome de inmunodeficiencia adquirida (SIDA) y la infección por el virus de la inmunodeficiencia humana (VIH) se ha convertido en una enfermedad crónica, con una mayor supervivencia y, concomitantemente, una mayor incidencia de enfermedades no relacionadas con el SIDA^(1)^. El control de la enfermedad con fármacos antirretrovirales forma parte del objetivo 95-95-95, que pretende que el 95% de las personas que viven con el VIH (PVV) conozcan su *estado serológico* en 2030, que el 95% estén en tratamiento antirretroviral y que el 95% tengan una carga viral suprimida^(2)^.

La atención a las PVV que antes se dirigía a las infecciones oportunistas se ha trasladado a otros problemas de salud que afectan a la población general^(3)^. Así, se considera que la adopción de prácticas de estilo de vida saludable, mediante una nutrición adecuada, ejercicio físico, no consumo de drogas lícitas e ilícitas, control del estrés y adherencia a los antirretrovirales y otros fármacos, es esencial para mejorar la calidad de vida de las personas que viven con el VIH^(4)^. Sin embargo, faltan incentivos para promover el autocuidado de un estilo de vida saludable entre estos pacientes^(5)^.

Los estudios muestran que, aunque las PVV tienen una mayor tasa de supervivencia con el TAR, las enfermedades crónicas no transmisibles (ENT) se dan con más frecuencia en esta población, en particular la enfermedad pulmonar obstructiva crónica, la cardiopatía isquémica, las enfermedades mentales y la disfunción renal y hepática^(6,7)^. El estilo de vida es un factor de aparición y mantenimiento de las ENT, y los cambios en este aspecto representan una intervención importante para prevenir las comorbilidades y hacer frente a las enfermedades^(8)^. Se necesitan herramientas asistenciales para el asesoramiento continuo de las PVV, así como intervenciones de educación en salud que fomenten la participación activa del paciente en el plan terapéutico^(9,10)^.

En vista de ello, las tecnologías han surgido como herramientas asistenciales facilitadoras capaces de difundir información precisa y conocimientos indispensables^(11)^. Los estudios demuestran que las tecnologías pueden ayudar a las PVV a mejorar su estilo de vida cambiando sus hábitos^(9,10)^. El avance de las tecnologías aporta innovaciones en el ámbito de la salud, sensibilizando a las PVV sobre el autocuidado, contribuyendo al acceso a la información y orientando a quienes necesitan servicios sanitarios^(5)^.

Las tecnologías en salud incluyen manuales, folletos, *carpetas*, *libros electrónicos*, programas educativos y *software* ^(12)^, que desempeñan un papel crucial en el desarrollo de acciones de prevención del VIH y en el seguimiento de los pacientes^(12)^. Las tecnologías en salud pueden clasificarse en blandas (tecnologías de relación, producción y comunicación, que implican acogida, vinculación y escucha); blandas-duras (conocimiento bien estructurado del proceso de salud, formulación de materiales educativos y aplicación de teorías); y duras (equipos tecnológicos, instrumentos, normas y*software*)^(13)^. Las tecnologías pueden ayudar a las enfermeras y a otros miembros del equipo sanitario multiprofesional en sus actividades de atención y orientación, con el fin de contribuir al autocuidado y a la adhesión a un estilo de vida saludable por parte de las PVV^(9)^. Sin embargo, antes de que puedan ser utilizadas por los profesionales sanitarios y los pacientes, es preciso evaluar su eficacia.

Las tecnologías en salud eficaces para la promoción de estilos de vida saludables colaboran tanto en la promoción de los cuidados por parte de los profesionales como en la autonomía y adherencia a estos cuidados por parte de las PVV. Evaluar si estas tecnologías son efectivas puede favorecer la práctica basada en la evidencia, dado que existen varios retos para implementar los cuidados en esta población, como la dificultad para acceder a los servicios de salud debido al estigma derivado de la enfermedad y al miedo a revelar el *estado serológico* ^(1,11)^, la baja alfabetización funcional en salud^(3)^, así como la baja escolarización e ingresos^(2,3,9,11)^. Todos estos aspectos pueden repercutir negativamente en la atención sanitaria y el estilo de vida.

Teniendo en cuenta lo anterior, este estudio fue guiado por la siguiente pregunta de investigación: ¿hasta qué punto son eficaces las tecnologías utilizadas para promover un estilo de vida saludable entre las PVV? Su objetivo fue analizar los ensayos clínicos que han evaluado la eficacia de las tecnologías para promover un estilo de vida saludable entre las personas que viven con el VIH.

## MÉTODO

### Tipo de Estudio

Se trata de una revisión sistemática, con un enfoque cuantitativo, descriptivo y sin metaanálisis, realizada en seis etapas: 1. Elaboración de la pregunta guía; 2. Formación de un protocolo; 3. Generación de una lista de estudios relevantes; 4. Selección de estudios para el análisis; 5. Evaluación de la calidad de los estudios y extracción de datos; 6. Síntesis y redacción del manuscrito^(14)^.

Se siguieron las recomendaciones del *Preferred Reporting Items for Systematic Reviews and Meta-Analyses* (PRISMA)^(15)^ y la revisión sistemática se registró en el *Registro Prospectivo Internacional de Revisiones Sistemáticas* (PROSPERO) en 2023, con el nº de registro CRD42023422772.

### Lugar y Período del Estudio

La búsqueda de artículos en las bases de datos se realizó entre octubre de 2023 y enero de 2024 por investigadores ubicados en Fortaleza, Ceará, Brasil, y Tumbes, Perú.

### Criterios de Elegibilidad

La pregunta de investigación se planteó según la estrategia PICO, acrónimo de Paciente (personas con VIH), Intervención (efectividad de las tecnologías de estilo de vida saludable), Comparación (no se aplicó en el estudio, ya que el objetivo no era comparar intervenciones) y Outcome (*Resultado)* (mejora del estilo de vida). La pregunta guía de la revisión sistemática fue: ¿cuál es la efectividad de las tecnologías de estilo de vida saludable para las PVV?

Los criterios de inclusión fueron estudios del tipo ensayo clínico aleatorizado, clasificados como nivel de evidencia II, que son ensayos clínicos controlados aleatorizados^(16)^, así como artículos completos disponibles en formato electrónico, sin restricciones de idioma o fecha de publicación, que incluyeran tecnologías de estilo de vida saludable para PVV mayores de 18 años. Los criterios de exclusión fueron los estudios con niños, adolescentes y mujeres embarazadas, así como los artículos repetidos, que se contabilizaron una sola vez.

### Fuentes de Información y Estrategias de Búsqueda en Bases de datos

Los artículos se seleccionaron de cinco bases de datos: *Medical Literature Analysis and Retrieval System Online* (MEDLINE); Scopus; Embase; *Web of Science*; y Latin American and Caribbean Health Sciences Literature (LILACS). Las bases de datos se eligieron en función de la pregunta orientadora, los tipos de estudios requeridos y su visibilidad en el área de la salud.

Fueron utilizados los descriptores «*HIV»*, «*HIV Infections* “, ”*Acquired Immunodeficiency Syndrome*», «*Technology*», «*Technologies»*, «*Life Style»* y «*Healthy Lifestyle* », todos de los Descriptores en Ciencias de la Salud (DeCS) de la Biblioteca Virtual en Salud y de los *Medical Subject Headings* (MeSH) de la *National Library of Medicine.* Los descriptores fueron reajustados de acuerdo con la localización de la búsqueda: insertados en inglés, seleccionando los operadores booleanos *AND* y *OR* en MEDLINE, Scopus y *Web of Science*; insertados en portugués, inglés y español, y el operador booleano *AND* en LILACS; insertados en inglés, seleccionando la opción con el operador booleano *AND* en Embase. Las estrategias de búsqueda y el número de artículos encontrados se describen en la Tabla 1.

**Tabla 1:** Estrategias de búsqueda de artículos en las bases de datos – Fortaleza, CE, Brasil, 2024.

Bases de datos	Cruzamientos en las bases de datos	Número de artículos
MEDLINE	("Acquired Immunodeficiency Syndrome"[Mesh] OR "HIV"[Mesh]) AND "Technology"[Mesh]	1.371
Scopus	(Technologies OR Technology) AND Lifestyle AND HIV	53
Embase	('Human Immunodeficiency Virus'/exp OR 'Human Immunodeficiency Virus' OR 'Acquired Immune Deficiency Syndrome'/exp OR 'Acquired Immune Deficiency Syndrome') AND ('Technology'/exp OR Technology) AND ('Lifestyle'/exp OR Lifestyle)	218
Web of Science	("HIV Infections" OR "Acquired Immunodeficiency Syndrome") AND (Technology OR Technologies)	841
LILACS	(HIV) AND (Technology) AND ("Healthy Lifestyle") OR ("Life Style")	196

### Proceso de Extracción y Análisis de la Información de los Estudios Seleccionados

Los artículos fueron seleccionados y analizados entre febrero y mayo de 2024 por dos revisores independientes, y se utilizó un tercero para definir los casos de desacuerdo entre los demás. Después de buscar en las bases de datos electrónicas, los artículos se exportaron a Rayyan, una herramienta *en línea* para construir revisiones sistemáticas^(17)^. Inicialmente, se eliminaron los estudios duplicados y, a continuación, se leyeron todos los títulos y resúmenes para identificar los estudios pertinentes, teniendo en cuenta los criterios de inclusión y exclusión. Los artículos en los que estos criterios no estaban claros se leyeron en su totalidad. A continuación, se evaluó la elegibilidad mediante la lectura completa de los estudios seleccionados. Los datos se extrajeron de los artículos y se organizaron en fichas clínicas con información sobre el título, la autoría, el año de publicación, el país del estudio, los objetivos, la muestra, los grupos de intervención y control, los resultados y los sesgos.

### Evaluación del Sesgo de los Estudios

Se utilizó la *Herramienta de Riesgo de Sesgo para Ensayos Aleatorios* (*Risk-of-Bias Tool For Randomized Trials*, RoB 2.0)^(18)^ para evaluar el sesgo de la investigación. La *Evaluación de Revisiones Sistemáticas Múltiples* (*Assessment of Multiple Systematic Reviews,* AMSTAR)^(19)^ se utilizó para evaluar la calidad de la revisión sistemática.

Los ocho artículos que respondían a la pregunta de investigación se analizaron mediante un enfoque organizado para ponderar el rigor y las características de los estudios, atendiendo al desarrollo metodológico, la intervención, la muestra, los resultados, la conclusión y el posible sesgo de la investigación. El riesgo de sesgo de los estudios se evaluó mediante la herramienta RoB 2.0 en cinco dominios: 1. sesgo derivado del proceso de aleatorización; 2. sesgo derivado de la desviación de la intervención prevista; 3. sesgo derivado de la falta de datos de los resultados; 4. sesgo derivado de la medición de los resultados; y 5. sesgo derivado de la selección de los resultados comunicados. Cada ámbito se evaluó como bajo riesgo de sesgo, alto riesgo de sesgo o motivo de preocupación^(18)^.

El AMSTAR evaluó la calidad de la revisión sistemática. Se trata de un instrumento con 16 ítems, que se corresponden con los requisitos mínimos de una revisión sistemática^(19)^: 1) ¿Incluían las preguntas de investigación y los criterios de inclusión de la revisión los componentes PICO? 2) ¿contenía el informe de la revisión una declaración explícita de que los métodos de revisión se establecieron antes de la revisión y justificaba el informe cualquier desviación significativa del protocolo? 3) ¿explicaban los autores de la revisión la selección de los diseños de estudio para su inclusión en la revisión? 4. ¿utilizaron los revisores una estrategia de búsqueda bibliográfica exhaustiva? 5. ¿eliminaron los revisores los estudios duplicados? 6) ¿Extrajeron los revisores datos por duplicado? 7. ¿proporcionaron los autores de la revisión una lista de los estudios excluidos y justificaron las exclusiones? 8. ¿describieron los autores de la revisión los estudios incluidos con el detalle adecuado? 9. ¿utilizaron los autores de la revisión una técnica satisfactoria para evaluar el riesgo de sesgo en los estudios individuales incluidos en la revisión? 10. 10) ¿Informaron los autores de la revisión de las fuentes de financiación de los estudios incluidos en la revisión? 11) Si se realizó un metanálisis, ¿utilizaron los autores de la revisión métodos apropiados para combinar estadísticamente los resultados? 12) Si se realizó un metanálisis, ¿evaluaron los autores de la revisión el impacto potencial del riesgo de sesgo en los estudios individuales sobre los resultados del metanálisis u otra síntesis de la evidencia? 13) ¿Tuvieron en cuenta los autores de la revisión el riesgo de sesgo en los estudios primarios al interpretar/discutir los resultados de la revisión? 14. ¿Los autores de la revisión proporcionaron una explicación y discusión satisfactorias de cualquier heterogeneidad observada en los resultados de la revisión? (15. Si llevaron a cabo una síntesis cuantitativa, ¿investigaron adecuadamente los autores de la revisión el sesgo de publicación (sesgo de estudios pequeños) y discutieron su probable impacto en los resultados de la revisión? 16. ¿Informaron los autores de la revisión sobre posibles fuentes de conflicto de intereses y sobre la financiación que recibieron para realizar la revisión?

Todos los ítems se responden con «sí» para los resultados positivos, «no» en los casos en que no se disponía de información o el revisor juzgó que no podía optar por el beneficio de la duda, o «parcialmente sí» cuando se consideró válido indicar la adhesión parcial al dominio. Siete de los 16 ítems son críticos (1, 4, 7, 9, 11, 13 y 15). Al final de la evaluación, la revisión se clasifica como críticamente baja (más de un fallo crítico), baja (un fallo crítico), moderada (más de un fallo no crítico) y alta (ningún fallo o un fallo no crítico)^(19)^. El AMSTAR fue aplicado de forma independiente por dos evaluadores, y las diferencias en las evaluaciones se discutieron y acordaron por consenso. Por último, se discutieron las conclusiones de los artículos de acuerdo con la literatura científica.

### Aspectos Éticos

En cuanto a los aspectos éticos, se respetaron los escritos de los artículos y los derechos de autor, sin modificar el contenido identificado, a favor de este estudio propuesto por los autores.

## RESULTADOS

Fueron identificados 2.679 artículos y, después del análisis, un total de ocho constituyeron el estudio. La figura 1 muestra el diagrama de flujo con el número de artículos seleccionados.

**Figura 1:** Diagrama de flujo de selección de estudios, adaptado de *Preferred Reporting Items for Systematic Reviews and Meta-Analyses* – Fortaleza, CE, Brasil, 2024.

El año de publicación de los artículos osciló entre 2006 y 2023, siendo tres realizados en Estados Unidos, dos en Brasil, uno en Canadá, uno en Vietnam y uno en China^(3,11,20–25)^. La caracterización de los artículos en cuanto a título, país de estudio, objetivos, muestra, grupos y resultados se muestra en la Tabla 2.

**Tabla 2:** Estudios sobre tecnologías de estilo de vida saludable para personas que viven con el VIH según título, país de estudio, objetivos, muestra, grupos y resultados – Fortaleza, CE, Brasil, 2024.

Título/país de realización del estudio	Objetivos	Muestra	Grupo intervención	Grupo control	Resultados
Using motivational interviewing to promote adherence to antiretroviral medications: a randomized controlled study^(20)^/Estados Unidos	Evaluación de la entrevista motivacional para mejorar la adherencia al TAR en PVV	Total: 247 GI: 125 GC: 122	Entrevista motivacional presencial, realizada por una enfermera en cinco sesiones de 20-90 minutos, a lo largo de tres meses en persona y por teléfono.	La norma de atención de la institución.	La GI fue superior a la GC en la adherencia al TAR seis meses después del inicio de la intervención (p < 0,001).
A feasibility study to develop and test a cognitive behavioral stress management mobile health application for HIV-related fatigue^(21)^/Estados Unidos	Determinar la viabilidad y aceptabilidad de la aplicación *mHealth* para smartphones CBSM para tratar y gestionar el estrés y la fatiga en PVV.	Total: 30 GI: 15 GC: 15	Descargaron y utilizaron la CBSM *mHealth* en sus teléfonos inteligentes, y se les pidió que completaran un módulo por semana.	Descargaron la aplicación *Lifesum*, que es una aplicación genérica de estilo de vida saludable, y la usaban una vez a la semana.	GI: mayor reducción de la fatiga que el GC (diferencia media: 1,6; IC del 95%: 0,3-2,8). GI: mayor reducción de la depresión hasta la 5ª semana (diferencia media: 9,7; IC 95%: 1,1-18,4).
Knowledge, attitude and practice of people with HIV regarding a healthy lifestyle: clinical trial^(3)^/Brasil	Evaluación de la eficacia de un folleto sobre conocimientos, actitudes y prácticas para un estilo de vida saludable en PVV	Total: 144 GI: 70 GC: 74	Recibió el folleto en la consulta, lo leyó y se lo llevó a casa para leerlo cada quince días durante seis meses.	La norma de atención de la institución.	El folleto educativo mejoró el conocimiento, la actitud y la práctica de estilos de vida saludables entre las PVV.
Mobile health technology for improving symptom management in low income persons living with HIV^(23)^/Canadá	Examinar el impacto de una aplicación *mHealth* (*mVip*) con estrategias de autocuidado para los síntomas de PVV.	Total: 76 GI: 40 GC: 40	Se conectaban a *mVip* al menos una vez a la semana para evaluar los síntomas y recibir consejos de autocuidado.	Descargaron *mVIP* pero no recibieron ninguna estrategia de autocuidado.	GI: mejora en 12 de los 13 síntomas de la infección por VIH y en la adherencia al TAR (p = 0,017), en comparación con el GC.
Booklet for healthy lifestyle in people with HIV: a clinical trial^(11)^/Brasil	Evaluar la eficacia de un folleto educativo para promover un estilo de vida saludable y la adherencia al tratamiento antirretroviral en PVV.	Total: 174 GI: 70 GC: 74	Consulta médica rutinaria, lectura del folleto en el ambulatorio, llevarse el folleto a casa para leerlo quincenalmente durante seis meses.	Consulta médica de rutina.	Al inicio del estudio, el estilo de vida de la mayoría de las PVV era insatisfactorio. El folleto mejoró el estilo de vida y el cumplimiento de la terapia antirretrovírica en GI, en comparación con la línea de base y el GC.
Efficacy of a mobile phone-based intervention on health behaviors and HIV/AIDS treatment management: randomized controlled trial^(24)^/Vietnam	Evaluar la viabilidad y la eficacia de la intervención *de salud móvil* en la adherencia al tratamiento antirretroviral, la autoeficacia y el comportamiento sanitario de las PVV en Vietnam.	Total: 425 GI: 238 GC: 187	Consultas periódicas sobre VIH/sida, uso de la app Ecare y seguimientos de uno y tres meses.	Consultas periódicas por VIH/sida. Seguimiento al mes y a los tres meses.	El GI aumentó la adherencia al TAR al cabo de uno y tres meses, en comparación con el GC. La mejora de las conductas de riesgo (tabaquismo, drogas y alcohol) fue positiva en el GI, pero limitada.
A randomized trial of a proactive cellular telephone intervention for smokers living with HIV/AIDS^(22)^/Estados Unidos	Evaluar la eficacia de una intervención para PVV para dejar de fumar.	Total: 95 GI: 48 GC: 47	Asesoramiento médico estándar e intervención telefónica, con ocho sesiones proactivas de asesoramiento presencial.	Asesoramiento estándar para dejar de fumar.	El GI tenía 3,6 veces más probabilidades de dejar de fumar en comparación con el GC (p = 0,0059). Las tasas de abstinencia fueron del 10,3% en el GC y del 36,8% en el GI.
Effect of a WeChat-Based Intervention (Run4Love) on depressive symptoms among people living with HIV in China: randomized controlled trial(25)/China	Evaluar la eficacia de una intervención *basada en WeChat* (*Run4Love*), con un ensayo clínico aleatorizado de 300 PVV con depresión en China.	Total: 300 PVV con depresión GI: 150 GC: 150	Recibieron el programa *Run4Love* con un curso sobre gestión de la reducción del estrés y afrontamiento, artículos breves sobre autocuidado y un programa de promoción de la actividad física, con objetivos fijados y *feedback* personalizado.	Recibieron un documento sobre nutrición y cuidados habituales para PVV en *WeChat*.	En comparación con el GC, el GI redujo la gravedad de la depresión (23,9 a 17,7 frente *a* 24,3 a 23,8), mejoró la calidad de vida (77,4 a 82,6 *frente a* 76,6 a 77,0), redujo el estrés (20,0 a 15,7 *frente a* 20,7 a 18,9) y mejoró el enfrentamiento (18,4 a 20,7 *frente a* 18,3 a 17,8).

Notas: TAR: terapia antirretrovírica; PVV: personas que viven con el VIH; GI: grupo de intervención; GC: grupo de control; IC: Intervalo de confianza.

Los estudios han evaluado diferentes tecnologías para promover estilos de vida saludables en PVV. Las tecnologías más utilizadas fueron aplicaciones móviles^(21,23–25)^, folletos impresos^(3,11)^, entrevistas motivacionales^(20)^ e intervenciones telefónicas^(20,22)^. Las tecnologías se utilizaron para promover la adherencia al TAR^(11,20,24)^, la salud mental, con prácticas para gestionar el estrés, la fatiga y la depresión^(21,25)^, fomentar el abandono y la reducción del tabaquismo^(22,24)^y reducir conductas de riesgo como el consumo de alcohol y drogas^(24)^. También se identificaron estudios que fomentaban el autocuidado^(3,11,23,25)^ y aspectos de un estilo de vida saludable, como la nutrición, el ejercicio físico, el comportamiento preventivo, las relaciones y la gestión del estrés^(3,11,25)^.

Todas las tecnologías resultaron eficaces para mejorar el estilo de vida de las PVV^(3,11,20–25)^. En cuanto a la clasificación de los tipos de tecnología^(13)^, los estudios incluyeron tecnologías blandas y duras^(3,11,20)^ y tecnologías duras^(20–25^). Las tecnologías blandas-duras incluían folletos educativos para promover un estilo de vida saludable, con orientación sobre el control del peso corporal, la alimentación saludable, el ejercicio físico, el control/abandono del tabaquismo y el consumo de alcohol/otras drogas, la gestión del estrés y la adherencia al TAR^(3,11)^, así como entrevistas motivacionales cara a cara para la adherencia al TAR^(20)^. Las tecnologías duras fueron las aplicaciones móviles^(21,23–25)^ y el asesoramiento telefónico^(20,22)^. Las aplicaciones móviles tenían como objetivo mejorar la gestión del estrés^(21,25)^ y la fatiga^(21)^, la adherencia al TAR^(23,24)^, la promoción del autocuidado^(23,25)^, el cambio de conductas de riesgo en el consumo de tabaco, alcohol y drogas^(24)^, la promoción de la actividad física y la reducción de la depresión^(25)^. Las intervenciones telefónicas promovieron la adherencia al TAR mediante entrevistas motivacionales(20^)^ y el abandono del tabaco^(22)^.

Al evaluar el riesgo de sesgo, según RoB 2.0^(18)^, se consideró que los ocho estudios presentaban cierto riesgo de sesgo, lo que se muestra en la Tabla 3.

**Tabla 3:** Evaluación del riesgo de sesgo de los estudios según la*herramienta Risk-of-Bias Tool For Randomised Trials* ^(18)^ – Fortaleza, CE, Brasil, 2024.

Riesgo de sesgo en los estudios	Artículos evaluados							
	(20)	(21)	(3)	(23)	(11)	(24)	(22)	(25)
1. Sesgo resultante del proceso de aleatorización	+	−	+	+	+	+	+	?
2. Sesgo debido a la desviación de la intervención prevista	+	+	?	+	?	?	+	?
3. Sesgo debido a la falta de datos de resultados	+	+	+	+	?	+	+	+
4. Sesgo derivado de la medición de los resultados	?	+	+	?	+	+	?	+
5. Sesgo derivado de la selección de los resultados comunicados	+	+	+	−	+	+	+	+
Otros sesgos	+	+	+	+	+	+	+	+

Clave: (+) riesgo bajo; (−) riesgo alto; (?) cierta preocupación.

La tabla 4 ofrece una descripción detallada de los sesgos en cada uno de los artículos analizados. Sobre todo, se destacan los sesgos relacionados con los aspectos metodológicos de los estudios.

**Tabla 4:** Descripción de los sesgos de los estudios según la herramienta Risk-of-Bias Tool For Randomised Trials^(18)^. Fortaleza, CE, Brasil, 2024.

Autoria de los estudios	Sesgos de los estudios de acuerdo con *Risk-of-Bias Tool For Randomized Trials*(18)
Dilorio et al., 2008^(20)^	La mayor parte de la muestra estaba compuesta por hombres afroamericanos con bajos ingresos, lo que impide generalizar los resultados. Se incluyó a pacientes sin mala adherencia al TAR. Dificultad para adherirse a la tecnología de recuento de pastillas, que consistía en un dispositivo acoplado al tapón del frasco.
Barroso et al., 2020^(21)^	Diferencias significativas entre los grupos, con predominio de hombres y personas con estudios superiores en el GC. Había tres personas en programas de recuperación de drogas ilícitas en el GI y ninguna en el GC.
Lima et al., 2022^(3)^	Mayor número de desempleados en el GC. Parte del estudio tuvo lugar durante la pandemia de COVID-19 en 2020, lo que puede interferir en los resultados debido al aislamiento social.
Schnall et al., 2018^(23)^	Los investigadores no especificaron los componentes, la organización del equipo de estudio ni si los evaluadores conocían al grupo que recibió la intervención.
Lima et al., 2023^(11)^	Había más desempleados en el GC. Parte del estudio tuvo lugar durante la pandemia de COVID-19 en 2020, lo que puede interferir en los resultados debido al aislamiento social de los participantes.
Tran et al., 2017^(24)^	Hubo una diferencia en el número de participantes en los grupos, que fue menor en el GC.
Vidrine et al., 2006^(22)^	No dijeron si los evaluadores de resultados conocían la intervención aplicada, si había un guión o un procedimiento operativo estándar para aplicar las intervenciones.
Guo et al., 2020^(25)^	El equipo de recolección de datos y los participantes en la intervención no estaban cegados. No se informó de si los instrumentos de evaluación de la calidad de vida o la depresión se aplicaron en línea o en persona en los meses siguientes. No se abordó el número de mujeres en el estudio, sólo el de hombres, homosexuales o bisexuales.

Notas: TAR: terapia antirretroviral; GC: grupo de control; GI: grupo de intervención; COVID-19: coronavirus-19.

Al evaluar la revisión sistemática mediante AMSTAR^(19)^, la calificación de la calidad metodológica se consideró alta, ya que sólo una de las 16 preguntas respondidas fue «no» cuando se preguntó si los autores de la revisión informaban de las fuentes de financiación de los estudios incluidos. Sin embargo, este ítem se considera un aspecto no crítico a evaluar, por lo que no compromete la calidad de esta revisión sistemática.

## DISCUSIÓN

La infección por VIH ha evolucionado de una enfermedad mortal a una condición crónica gracias a la terapia antirretroviral, por lo que los afectados tienen una mayor supervivencia, exponiéndose a las condiciones sociales, psicológicas, biológicas, culturales y espirituales asociadas a la enfermedad, así como a los eventos adversos a largo plazo de los medicamentos antirretrovirales^(23)^. Las investigaciones también han observado que esta población tiene un estilo de vida poco saludable, asociado a un estilo de vida sedentario, dieta inadecuada, tabaquismo, consumo de alcohol y otras drogas, así como ansiedad, depresión y fatiga^(11,21,24)^.

Este estudio reunió artículos sobre las tecnologías existentes para mejorar el estilo de vida de las PVV. Destacaba el uso de aplicaciones móviles^(21,23,24,25)^, folletos impresos^(3,11)^, entrevistas motivacionales e intervenciones telefónicas^(20,22)^. Las tecnologías estaban dirigidas a la adherencia antirretroviral, el manejo del estrés y la fatiga, el ejercicio físico, la disuasión del tabaquismo, así como otros aspectos para promover un estilo de vida saludable.

Los estudios han utilizado predominantemente aplicaciones móviles, denominadas tecnologías duras^(13,21,23,24,25)^. Las aplicaciones móviles destacan por su facilidad de acceso en cualquier momento o lugar, lo que favorece la adherencia en el ámbito de la salud^(25)^. Sin embargo, una de las dificultades es que la mayoría de las apps requieren un teléfono móvil e internet para acceder a los contenidos, lo que puede suponer un problema para poblaciones económicamente vulnerables^(25,26)^. Las apps forman parte de las tecnologías sanitarias móviles (*mHealth*) y permiten intervenciones basadas en dispositivos, lo que las hace convenientes para la educación en salud^(27)^. Aunque tienen el potencial de mejorar los estilos de vida de las personas que viven con el VIH, en muchas de ellas no se ha evaluado su eficacia clínica y se necesitan estudios con este fin^(23)^.

La aplicación *Cognitive Behavioural Stress Management* (CBSM) fue una intervención educativa para la gestión del estrés, las habilidades interpersonales, la relajación, la resolución de problemas y las estrategias de afrontamiento^(21)^. eCARE, por su parte, pretendía reducir los comportamientos de riesgo de las PVVS, como el tabaquismo y el consumo de alcohol y otras drogas^(24)^. Las aplicaciones en el contexto de *la sanidad móvil* pueden ayudar a las PVVS que viven lejos de las instituciones sanitarias a acceder a información que no estaría fácilmente disponible, así como facilitar la comunicación con grupos minoritarios^(21,23,25)^.

Un estudio realizado en China incorporó la CBSM a una red social para proporcionar múltiples seguimientos a largo plazo. Redujo significativamente la gravedad de la depresión, mejoró la calidad de vida, tuvo una buena viabilidad y un alto nivel de satisfacción de los usuarios^(25)^. Sin embargo, una desventaja observada en el uso de las apps es la dependencia de los pacientes a la hora de gestionar su estado de salud y tratamiento únicamente a través de la app, por lo que se necesitan estudios que evalúen y propongan soluciones a esta cuestión^(24)^.

Dadas las desventajas citadas en el uso de aplicaciones móviles, está claro que también se necesitan tecnologías sanitarias que no utilicen teléfonos o Internet, como folletos que puedan imprimirse y distribuirse, como se analiza en dos estudios de esta revisión^(3,11)^. Este tipo de tecnología puede ser entregada por los profesionales sanitarios a las PVVS durante las consultas o la educación sanitaria, pero la dificultad para conseguir el efecto deseado es la necesidad de que el usuario sea capaz de leer e interpretar los textos^(3,11)^.

Cabe destacar que es necesario conocer el impacto de estas tecnologías en la vida de los pacientes, así como el grado de importancia que tienen para el autocuidado, ya que no todos los individuos están preparados para recibir determinadas tecnologías. Por ejemplo, el hecho de que se distribuyan folletos o accesos a apps y algunos pacientes no sepan leer o no dispongan de la tecnología o dispositivo necesario para acceder al contenido, puede llevar a situaciones embarazosas que incluso impliquen aspectos éticos. Por otro lado, las tecnologías deben preparar a los pacientes para la autonomía y la independencia mediante la práctica del autocuidado. Teniendo en cuenta esto, las tecnologías deben desarrollarse con miras a una mayor inclusión y acceso para los diferentes tipos de usuarios, como el uso de tecnologías adaptadas, con la aplicación de vídeo y sonido, de una manera más completa, que a su vez depende de las políticas públicas actuales, la democratización de la educación, la distribución de ingresos, así como el libre acceso a Internet^(23)^.

El folleto titulado «¡Mi cartilla para motivar el cambio! Prácticas para promover un estilo de vida saludable» fue una tecnología validada que mostró mejoras en los conocimientos, actitudes y prácticas de estilo de vida en PVV a los dos, cuatro y seis meses de la intervención, en comparación con el grupo de control^(3,11)^. Los folletos son una tecnología de bajo coste, fácil de manejar, fácil de entender y fácil de llevar que tiene un impacto positivo y genera cambios en el comportamiento^(28)^.

Las intervenciones tecnológicas en salud son importantes para las PVV y representan una atención continuada y adicional por parte de los profesionales sanitarios que va más allá de la consulta^(11)^. A pesar de la variedad de materiales educativos disponibles, los folletos adoptan un enfoque objetivo de la información y las prácticas de autocuidado, con un lenguaje fácil de entender, lo que favorece los comportamientos positivos en las PVV y mejora el vínculo con el equipo de salud^(11,29)^. Ya se han tratado varios temas en folletos para PVV, como la salud sexual y reproductiva para parejas serodiscordantes^(30)^, la prevención de la transmisión maternoinfantil del VIH^(28)^, así como temas generales sobre promoción de la salud, como la lucha contra el estigma y la discriminación, los derechos de las PVV y la salud física^(29)^.

Otros dos estudios utilizaron la entrevista motivacional y la telesalud, ambas a través de llamadas telefónicas, que se consideran tecnologías duras^(13,20,22)^. Aunque las aplicaciones móviles han ido ganando terreno en los últimos años, las intervenciones telefónicas son una buena estrategia para llegar a las personas que no tienen acceso a Internet o que no saben utilizar las aplicaciones. Sin embargo, se necesita tiempo y ubicación para recibir llamadas, ya que algunas PVV sufren estigmatización y no comparten su diagnóstico, por lo que no pueden atender llamadas en público y necesitan un entorno privado^(31)^.

La entrevista motivacional promueve la colaboración entre los profesionales sanitarios y los pacientes y se centra en la motivación para el cambio, el respeto a la autonomía, la empatía y el compromiso profesional^(32)^. También resultó eficaz en otro estudio con PVV, en el que se produjo una reducción del 34% en el consumo de alcohol, con repercusiones en la salud y una mejora del estilo de vida^(33)^. Esta tecnología también se ha utilizado para reducir el consumo de drogas y el riesgo sexual en un estudio piloto con 50 parejas masculinas, en el que se redujo el sexo anal sin preservativo y el consumo de drogas ilícitas^(34)^.

A pesar del gran número de estudios identificados sobre el tema, cuando los materiales fueron leídos en profundidad, la investigación predominante fue para la construcción y validación de tecnologías, sin ensayos clínicos, lo que limitó la muestra de artículos en esta revisión. Además, una limitación de esta revisión sistemática fue la imposibilidad de realizar un meta-análisis con base en los estudios seleccionados, pues las características metodológicas de los ensayos clínicos imposibilitaron el cálculo de medidas de resumen.

Para futuros estudios, se sugiere que las tecnologías se apliquen en ensayos clínicos aleatorizados para evaluar la eficacia a largo plazo de las intervenciones, y que estos estudios sigan estrictamente las *normas CONS* ORT (*CONsolidated Standards Of Reporting Trials*) para reducir el sesgo. Además, es importante aplicar estas tecnologías a PVV de diferentes regiones geográficas para diversificar las poblaciones analizadas, así como evaluar los costes y la viabilidad de su implantación en servicios sanitarios, poblaciones o comunidades.

## CONCLUSIÓN

Las tecnologías utilizadas para promover estilos de vida saludables en PVV han demostrado ser eficaces para la adherencia al TAR, el manejo del estrés, la promoción del autocuidado, la reducción del tabaquismo y las conductas de riesgo, destacando el uso de apps, folletos y llamadas telefónicas. La implantación de tecnologías sanitarias va en aumento, por lo que se hace necesario ampliar estos recursos entre las PVV, una población con necesidades centradas no sólo en la infección, sino también en la promoción de un estilo de vida saludable, con el fin de prevenir otras patologías crónicas derivadas del envejecimiento, de la propia infección por VIH y del uso prolongado del TAR.
